# Multifaceted Integrated Analysis of CDK1 and TOP2A Signaling Pathways for Multi-Target Therapeutic Intervention in Epithelial Ovarian Cancer

**DOI:** 10.3390/ijms27125264

**Published:** 2026-06-10

**Authors:** Saber Samadiafshar, Mahla Masoudi, Hossein Azizi, Thomas Skutella

**Affiliations:** 1 Pediatric Health Research Center, Tabriz University of Medical Sciences, Tabriz 5143377505, Iran; sabersamadiafshar@yahoo.com; 2 Department of Stem Cells and Cancer, College of Biotechnology, Amol University of Special Modern Technologies, Amol 4615863111, Iran; mahlamasoudii@gmail.com; 3 Institute for Anatomy and Cell Biology, Medical Faculty, University of Heidelberg, Im Neuenheimer Feld 307, 69120 Heidelberg, Germany

**Keywords:** epithelial ovarian cancer, CDK1, molecular dynamics simulations, TOP2A, Protein–Protein Interaction (PPI)

## Abstract

Epithelial ovarian cancer (EOC) remains one of the most aggressive gynecological malignancies, largely due to late-stage diagnosis, therapeutic resistance, and molecular heterogeneity. This study aimed to identify biologically relevant hub genes and evaluate potential dual-target compounds against *Cyclin-Dependent Kinase 1 (CDK1)* and *DNA Topoisomerase II Alpha (TOP2A)* through an integrated computational framework. Transcriptomic datasets from GSE28799, GSE54388, and GSE14407 were analyzed to identify overlapping differentially expressed genes, followed by protein–protein interaction analysis, functional enrichment, survival assessment, molecular docking, ADMET profiling, and molecular dynamics simulations. Mechanistically, CDK1 and TOP2A participate in coordinated cell-cycle regulation associated with G2/M progression and chromosomal dynamics in ovarian cancer. Among the identified hub genes, *CDK1* and *TOP2A* demonstrated marked overexpression and central topological importance within the interaction network. Functional enrichment analyses highlighted significant associations with mitotic cell-cycle regulation, DNA replication, and proliferative signaling pathways. Molecular docking analyses identified Naringin as a potential dual-target candidate with favorable binding affinity toward both CDK1 and TOP2A. ADMET profiling suggested acceptable pharmacokinetic and toxicity characteristics, while molecular dynamics simulations supported stable protein–ligand interactions under dynamic conditions. Although survival analyses did not demonstrate statistically significant independent prognostic associations, the findings support the biological relevance of *CDK1* and *TOP2A* in EOC progression. Collectively, this study provides an integrated computational perspective on CDK1/TOP2A-associated oncogenic signaling and prioritizes Naringin as a preliminary candidate for future experimental investigation in epithelial ovarian cancer.

## 1. Introduction

Ovarian cancer remains one of the most lethal gynecological malignancies worldwide, presenting formidable challenges across all dimensions of clinical management [1]. These challenges encompass late-stage diagnosis at presentation, the development of chemoresistance despite adherence to standard-of-care therapeutic protocols including cytoreductive surgery and platinum-based adjuvant chemotherapy, elevated recurrence rates following initial treatment observed in approximately 60% of cases, and the persistently dismal long-term prognoses associated with advanced disease [2]. On a global scale, ovarian cancer ranks fifth among all cancers affecting women, with a staggering 70% of cases progressing to advanced FIGO stages III and IV at the time of diagnosis, a phenomenon largely attributable to the anatomically concealed position of the ovaries within the pelvic cavity, which impedes early detection efforts [3,4]. The consequent diagnostic delay frequently reveals the presence of distant metastases, substantially restricting the efficacy of therapeutic interventions and contributing to elevated mortality rates [5]. In this context, systematic exploration of the novel molecular pathways implicated in ovarian cancer pathogenesis holds considerable promise for unveiling innovative diagnostic and therapeutic strategies to address this formidable disease. Given the molecular heterogeneity of epithelial ovarian cancer and the limited durability of current therapeutic strategies, identification of interconnected regulatory targets involved in tumor progression remains a major research priority.

The ovaries serve as vital organs for female reproduction and fertility. Although only 12% of identified ovarian cancer cases occur during the reproductive years or before the age of 44, this statistic nonetheless reflects the significant impact of ovarian cancer on fertility and infertility rates among affected individuals and couples [6]. From a histopathological perspective, ovarian cancers predominantly originate from surface epithelial cells in approximately 90% of cases and are further subclassified based on histological features into serous (approximately 68%), endometrioid (11%), low-grade serous carcinoma (3%), mucinous (3%), and clear cell carcinoma (12%) subtypes [7]. Underlying this malignant transformation, somatic gene mutations and their downstream functional cascades leading to uncontrolled cellular proliferation play a fundamental role in tumor formation and progression [8]. The drawbacks of conventional pharmacological therapies, including dose-limiting side effects and the emergence of drug resistance, underscore the urgent need to identify novel targetable cellular vulnerabilities and to develop anti-tumor compounds characterized by high efficacy and low systemic toxicity [9]. Since the early 1970s, the discovery of cell cycle regulatory mechanisms has highlighted the pivotal role of cell cycle checkpoints in tumor biology and drug research [8]. Within the in silico paradigm, target proteins are assessed alongside small molecule inhibitors to evaluate molecular interactions and binding affinities, constituting a systematic and resource-efficient framework for drug discovery and efficacy enhancement [10]. Recent computational and molecular pharmacology studies have similarly emphasized the therapeutic relevance of multi-target strategies involving cell-cycle regulatory proteins in ovarian malignancies [11].

In the context of EOC, molecular docking investigations in the present study were focused on CDK1 and TOP2A, two proteins that influence distinct but functionally interconnected cell cycle checkpoints: TOP2A at the S/G2 checkpoint and CDK1 at the G2/M checkpoint. Deciphering drug resistance mechanisms and facilitating the discovery of therapeutic agents with robust multi-target efficacy are of paramount importance in this field. Unlike previous studies primarily focused on isolated CDK1-targeting strategies, the present investigation integrates transcriptomic profiling, network topology analysis, dual-target pharmacological evaluation of CDK1 and TOP2A, ADMET prioritization, and molecular dynamics simulations within a unified epithelial ovarian cancer framework.

Molecular docking has emerged as a powerful computational technique for characterizing the intricate interactions between small molecule inhibitors and their target proteins. However, as docking provides a static representation of protein–ligand interactions, molecular dynamics simulations are increasingly employed to evaluate the stability and dynamic behavior of these complexes under physiologically relevant conditions. Complementing these approaches, AdmetSAR provides highly accurate predictions of pharmacokinetic and toxicological properties, thereby offering a comprehensive and systematic platform for drug discovery, lead optimization, and validation of candidate compounds. The present study advances the understanding and potential treatment of EOC through several innovative methodological contributions. By integrating comprehensive microarray analyses with protein–protein interaction network construction, CDK1 and TOP2A were identified and validated as key molecular determinants of EOC progression. The application of in silico techniques, including molecular docking, molecular dynamics simulations, and ADMET profiling, enhanced the precision with which effective therapeutic candidates were identified. Notably, Naringin emerged as a particularly promising natural compound, exhibiting favorable binding properties and a minimal side-effect profile, thereby underscoring the broader therapeutic potential of phytochemical agents in oncology. Following an extensive investigation of the functional pathway contributions of these genes across multiple databases, molecular docking simulations were conducted to explore the binding affinities and interaction profiles of 29 diverse drug candidates with the TOP2A and CDK1 proteins. These compounds include Alsterpaullone, Avotaciclib, Fostamatinib, Naringin, Amsacrine, Dexrazoxane, Valrubicin, Teniposide, Etoposide, Doxorubicin, Idarubicin, Mitoxantrone, Epirubicin, Podofilox, Genistein, Ciprofloxacin, Enoxacin, Fleroxacin, Lomefloxacin, Moxifloxacin, Norfloxacin, Pefloxacin, Sparfloxacin, Trovafloxacin, Ofloxacin, Lucanthone, Daunorubicin, Finafloxacin, and Aldoxorubicin. This multi-compound approach provides a strategic framework for the discovery of multi-targeted pharmacological agents, with the overarching goals of addressing chemoresistance mechanisms and improving clinical outcomes for patients with EOC. Furthermore, molecular dynamics simulations were conducted to validate the stability of the top-performing compound, Naringin, in complex with both CDK1 and TOP2A, providing dynamic insights into its binding behavior and reinforcing its potential as a dual-target interaction requiring further experimental validation.

## 2. Results

### 2.1. Gene Expression

**Figure 1:**
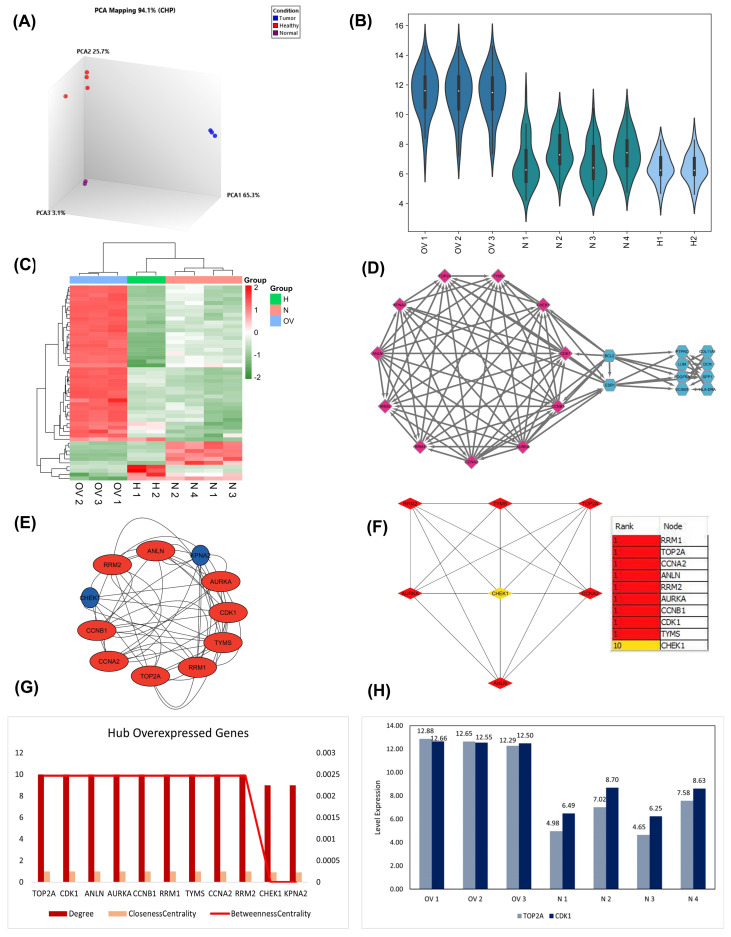
Integrated Analysis of Multivariate Data. (**A**) PCA plots indicate that biological replicates form distinct clusters; (**B**) Examining the data distribution in epithelial ovarian tumor (OV) and normal (N, H) groups using a violin plot; (**C**) The heatmap illustrates the disparity in hub gene expressions between the three compared groups. The zigzag pattern on a heat map indicates differential expression among genes; (**D**) Protein–protein interaction (PPI) network. In this visualization, upregulated genes are represented by red diamonds, while downregulated genes are depicted as blue hexagons; (**E**) PPI network based on betweenness centrality (size of genes) and degree parameters (red indicates higher degree and blue indicates lower degree); (**F**) The CytoHubba plugin 0.1 was used to apply the closeness centrality parameter to the network; (**G**) Comparative visualization of gene strength using box plots; (**H**) The expression levels of two hub genes across the analyzed groups. The genes *TOP2A* and *CDK1* exhibit the highest potency in driving ovarian epithelial cancer. Expression levels are presented as normalized transcript expression values [log2(TPM + 1)].

Following the retrieval and mining of gene expression profiles from epithelial ovarian cancer dataset GSE28799 and normal ovarian epithelial cell datasets GSE54388 and GSE14407, all available through the GEO repository, a comprehensive set of differentially expressed genes (DEGs) was defined. To visualize and interrogate the structural properties of the resulting high-dimensional data, a Principal Component Analysis (PCA) plot was generated (Figure 1A). This representation facilitated the identification of grouping patterns, inter-sample relationships, and potential outliers. Three distinct clustering patterns were observed, with data points exhibiting close spatial proximity within each cluster, indicative of consistent gene expression profiles across biological replicates within each group. To enable thorough cross-cohort comparisons and to explore the nuances of data distribution, a violin plot was subsequently constructed (Figure 1B). The ovarian epithelial cancer samples (OV) demonstrated a symmetrical distribution pattern, justifying the use of symmetric violin representations, whereas the normal tissue samples (N) displayed varying widths, reflecting different underlying data distribution tendencies. Given the approximate parity in overall data range across cohorts, the comparability of these groups for downstream analyses was affirmed.

By applying filtering criteria of Log2FC = 6 and a *p*-value threshold of 0.05, the gene list was refined to 21 genes, substantially enhancing the precision and interpretability of the analysis. The expression intensities of these genes across the study groups were visualized through a heatmap (Figure 1C), in which genes with LogFC > 0 were categorized as upregulated and those with LogFC < 0 were considered downregulated. Protein–protein interaction (PPI) relationships among the DEGs were identified using the STRING database and subsequently visualized using Cytoscape software 3.10.0 (Figure 1D). Building on these transcriptomic findings, network-level analyses were performed to map functional interactions among the dysregulated gene products.

Within the resulting network, *BCL2* and *ESR1* emerged as the most highly connected nodes among upregulated genes, functioning as intermediary hubs that modulate the influence of upregulated genes on downstream downregulated targets, thereby contributing to the inhibition of relevant signaling pathways. Given that upregulated genes exert a disproportionate influence on disease progression, these genes constituted the primary focus of the present analysis. The interactions among upregulated genes were re-examined using the STRING database, and gene-level topological parameters were applied within Cytoscape 3.10.0 (Figure 1E). The “degree” parameter, represented by color intensity, quantified the extent of each gene’s interactions within the network, while “betweenness centrality,” represented by node size, reflected the extent to which each gene serves as an information conduit. Closeness centrality was further assessed using the CytoHubba plugin 0.1 (Figure 1F), which revealed that, with the exception of *CHEK1* and *KPNA2*—which displayed comparatively lower centrality values—the remaining genes exhibited consistent average shortest path lengths to all other network nodes.

Through the integration of network topology visualization and comparative gene strength analysis via box plots, *TOP2A* and *CDK1* were identified as the most robustly upregulated genes in epithelial ovarian cancer progression (Figure 1G). Independent mRNA expression validation demonstrated marked overexpression of both *CDK1* and *TOP2A* in ovarian cancer tissues compared with normal controls, further supporting their biological relevance and consistency with the transcriptomic findings obtained from the integrated GEO datasets. To further investigate the biological relevance of these hub genes, functional enrichment analyses were subsequently conducted. Examination of their respective expression levels across comparison groups confirmed that both genes exhibit a similar upregulation pattern in cancer samples, while in control samples, *CDK1* expression was specifically elevated relative to *TOP2A* (Figure 1H).

**Figure 2:**
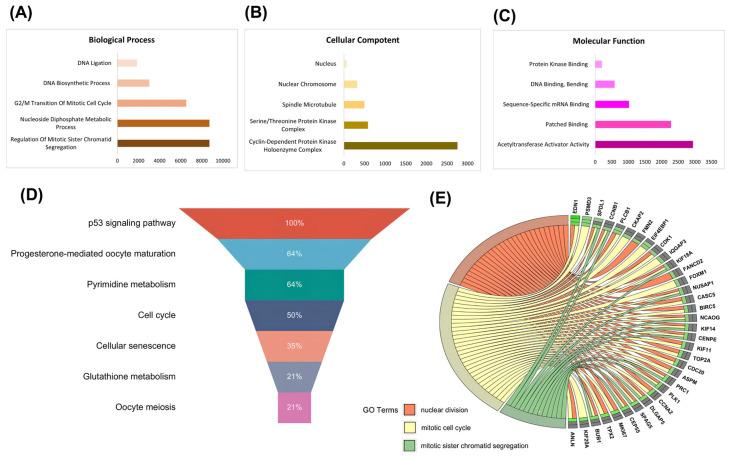
Gene Ontology and KEGG analysis of upregulated genes. (**A**) Biological Process; (**B**) Cellular Component; (**C**) Molecular Function; (**D**) KEGG Pathways (The numbers represent the enrichment percentages of the pathways); (**E**) Circular genome data visualization (circos) plot for the selected overrepresented Gene Ontology (GO) terms and corresponding differentially expressed genes (DEGs).

Gene ontology (GO) and pathway enrichment analyses were performed by submitting the gene list to the Enrichr database. The biological processes most significantly enriched included the regulation of mitotic sister chromatid segregation, nucleoside diphosphate metabolic processes, G2/M transition of the mitotic cell cycle, DNA biosynthetic processes, and DNA ligation (Figure 2A). At the cellular component level, the identified genes were associated with the Cyclin-Dependent Protein Kinase Holoenzyme Complex, Serine/Threonine Protein Kinase Complex, Spindle Microtubule, Nuclear Chromosome, and Nucleus (Figure 2B). In terms of molecular function, enriched activities included Acetyltransferase Activator Activity, Patched Binding, Sequence-Specific mRNA Binding, DNA Binding and Bending, and Protein Kinase Binding (Figure 2C). KEGG pathway analysis further revealed significant enrichment of the p53 Signaling Pathway, Progesterone-mediated oocyte maturation, Pyrimidine metabolism, Cell Cycle, Cellular Senescence, Glutathione metabolism, and Oocyte meiosis pathways (Figure 2D).

To further elucidate the functional interrelationships between key biological processes and the identified upregulated genes, a circular genome data visualization (circos) plot was generated (Figure 2E). This representation integrates selected overrepresented GO terms with their corresponding DEGs, providing an intuitive overview of gene–function associations within the network. The circos plot highlights three major enriched biological processes—nuclear division, mitotic cell cycle, and mitotic sister chromatid segregation—which are strongly and recurrently interconnected with a shared subset of upregulated hub genes. Notably, several genes, including *TOP2A, CDK1, KIF11, CENPE*, and *NUSAP1*, were found to be linked to multiple GO terms simultaneously, indicating their multifunctional roles in coordinating mitotic progression and chromosomal dynamics. The density and thickness of the connecting ribbons reflect the degree of gene involvement across biological processes, revealing a high degree of functional overlap among mitosis-related pathways. This convergence strongly suggests that the dysregulation of a core group of cell cycle-associated genes collectively drives aberrant cell division in epithelial ovarian cancer. Taken together, the circos analysis demonstrates that the upregulated genes do not act in isolation but rather constitute an integrated functional module centered on mitotic control, reinforcing the pivotal role of cell cycle dysregulation in ovarian cancer pathogenesis. Based on these findings, CDK1 and TOP2A were prioritized for subsequent computational pharmacological investigation. The prioritization of CDK1 and TOP2A was not based solely on network-ranking metrics. Instead, these genes were selected through an integrated evaluation framework incorporating differential expression magnitude, network connectivity, functional enrichment profiles, coordinated involvement in cell-cycle regulation, established relevance to ovarian cancer biology, and suitability for pharmacological targeting.

**Figure 3:**
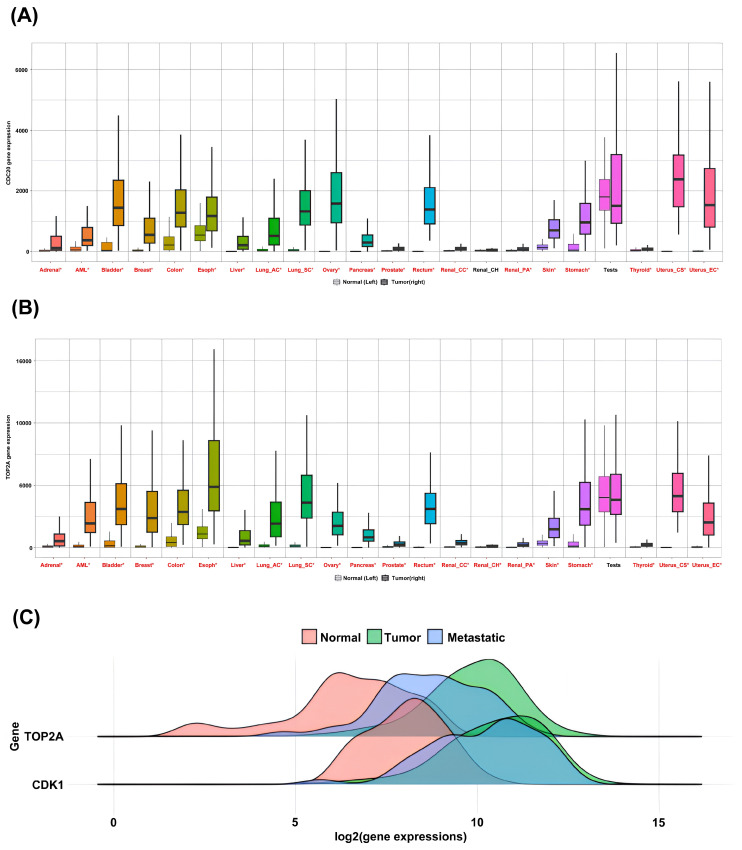
(**A**,**B**) Boxplot depicting the mRNA levels of *CDK1* and *TOP2A* in both normal and tumor human tissues. Tissue labels shown in red indicate a statistically significant difference in gene expression between normal and tumor samples (* *p* < 0.05, Mann–Whitney U test); (**C**) Comparison of the overlap of two gene expressions in three groups (metastatic, tumor, and normal) following multi-gene analysis. The highest overlap is observed in the tumor group.

The expression of both *TOP2A* and *CDK1* was found to be highly regulated across a broad spectrum of human malignancies, including uterine, esophageal, gastric, rectal, lung, liver, colorectal, basal cell, hematological, bone marrow, and breast cancers, underscoring their potential role in pan-cancer tumorigenesis (Figure 3A,B). Density plots were employed to compare the expression distributions of *TOP2A* and *CDK1* across normal, tumor, and metastatic groups (Figure 3C). Both genes exhibited a clear rightward shift in tumor samples relative to normal tissues, reflecting markedly increased expression in the cancerous state. The metastatic group showed substantial distributional overlap with the tumor group, suggesting maintenance of elevated expression throughout disease progression. In contrast, normal samples were concentrated at considerably lower expression ranges. The pronounced distributional overlap between *TOP2A* and *CDK1* expression profiles in tumor samples supports the notion of coordinated transcriptional regulation of these genes within the tumor microenvironment.

**Figure 4:**
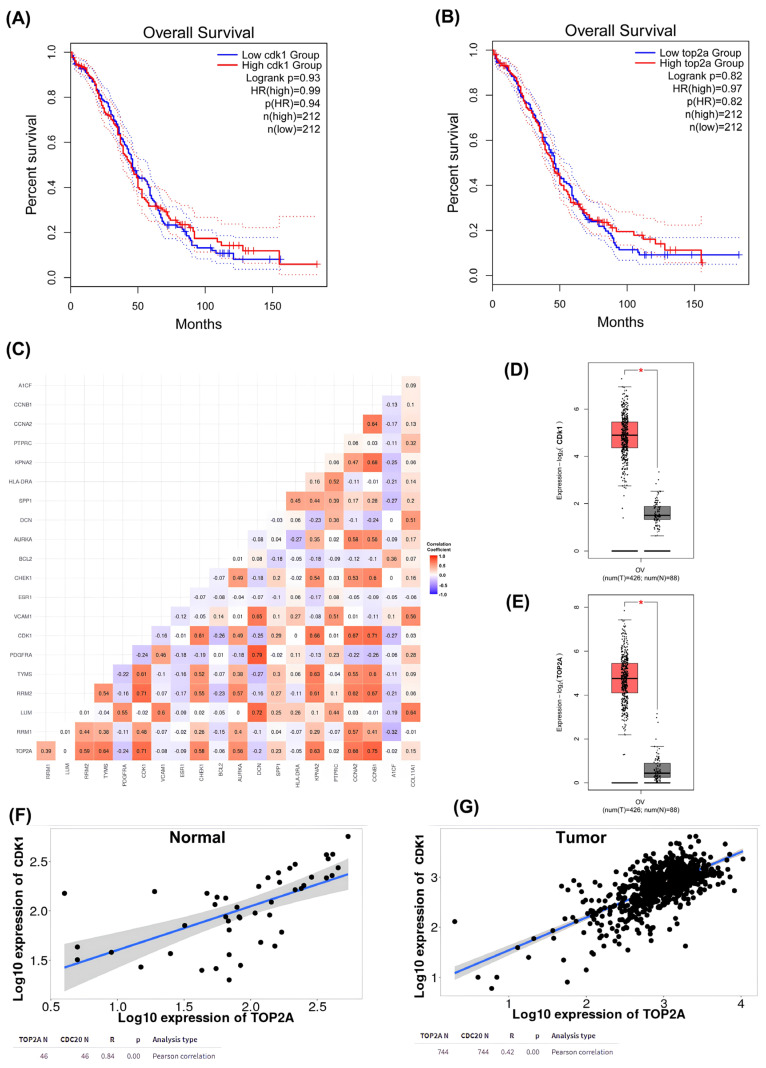
Clinical relevance of *CDK1* and *TOP2A* in ovarian cancer. Kaplan–Meier overall survival curves for high- and low-expression groups of *CDK1* (**A**) and *TOP2A* (**B**); (**C**) Evaluating Spearman correlation coefficient via a heatmap; Differential expression of *CDK1* (**D**) and *TOP2A* (**E**) between ovarian cancer and normal tissues based on TCGA and GTEx datasets, (*) indicates statistically significant difference (*p* < 0.05); (**F**,**G**) Using the Pearson coefficient, the scatter plot illustrates the correlation between two hub genes within the normal and tumor groups. A remarkable correlation was observed between the two genes *CDK1* and *TOP2A*.

Kaplan–Meier survival analysis was performed to evaluate the association between *CDK1* expression levels and overall survival, with patients stratified into high- and low-expression groups based on the median expression value. No statistically significant difference in overall survival was observed between the two groups, as evidenced by largely overlapping survival curves (log-rank *p* = 0.93; HR = 0.99), indicating that *CDK1* expression alone was not independently associated with patient survival in this cohort (Figure 4A). An analogous analysis performed for *TOP2A* expression likewise revealed no significant separation of overall survival curves between high- and low-expression groups, with the hazard ratio remaining close to unity (log-rank *p* = 0.82; HR = 0.97), suggesting that *TOP2A* expression in isolation does not significantly impact overall survival outcomes (Figure 4B).

A correlation heatmap was constructed to analyze the relationship matrix among DEGs within the tumor context, with correlation strength quantified using the Spearman correlation coefficient and represented through escalating color intensity (Figure 4C). The Spearman correlation coefficient between *TOP2A* and *CDK1* was determined to be 0.71, a positive value proximate to 1, indicative of a robust and statistically significant positive co-expression relationship between these two genes. This finding implies that increases in the expression of one gene are proportionally associated with increases in the expression of the other.

Boxplot analyses were subsequently performed to evaluate the differential expression of *CDK1* between ovarian tumor and normal tissue samples. The results demonstrated marked upregulation of *CDK1* in tumor tissues relative to normal samples, with a statistically significant difference confirmed by the Mann–Whitney U test (*p* < 0.05), indicating substantially enhanced *CDK1* expression in the tumor context (Figure 4D). Similarly, *TOP2A* expression analysis revealed a pronounced increase in tumor samples relative to normal tissues, with minimal distributional overlap between groups and a statistically significant difference (*p* < 0.05, Mann–Whitney U test), confirming the strong overexpression of *TOP2A* in ovarian cancer specimens (Figure 4E).

Scatter plot analyses were performed to assess the strength and directionality of the linear relationship between *CDK1* and *TOP2A* in both normal and tumor groups. In the normal group, the scatter plot revealed dispersed data points with a positive slope, reflecting a robust positive correlation between the two genes, further supported by a high Pearson R-value of 0.84 (Figure 4F). This strong co-expression suggests that alterations in one gene correspond closely with changes in the other under physiological conditions. In the tumor group, despite also displaying a positively inclined regression line, data points were noticeably more tightly clustered, and the Pearson correlation coefficient was comparatively lower at R = 0.42, indicative of a weaker positive correlation that may reflect the influence of additional molecular factors within the tumor microenvironment (Figure 4G). The consistently significant *p*-values across both groups confirm the statistical robustness of the observed correlations.

### 2.2. In Silico Simulation

#### 2.2.1. Molecular Docking Simulation

TOP2A represents a well-established pharmacological target in human cancer, with its functional pathway rendering it susceptible to inhibition by a range of anticancer compounds. In parallel, CDK1 plays an indispensable role in cancer cell cycle control, both through its direct influence on the G2/M transition and through its regulatory relationship with TOP2A, collectively forming the dual focus of the present in silico investigation. Table 1 summarizes the molecular docking results obtained from AutoDock Vina 1.2.x, detailing the binding affinity values (in kcal/mol) and binding site coordinates for both CDK1 and TOP2A proteins. A complementary section of Table 1 presents the interaction analysis output from the PLIP server, encompassing hydrophobic interactions, hydrogen bonds, and salt bridges derived from the docking outputs. Together, these analyses provide detailed insights into the binding modes and interaction profiles of the evaluated compounds with the CDK1 and TOP2A target proteins.

**Figure 5:**
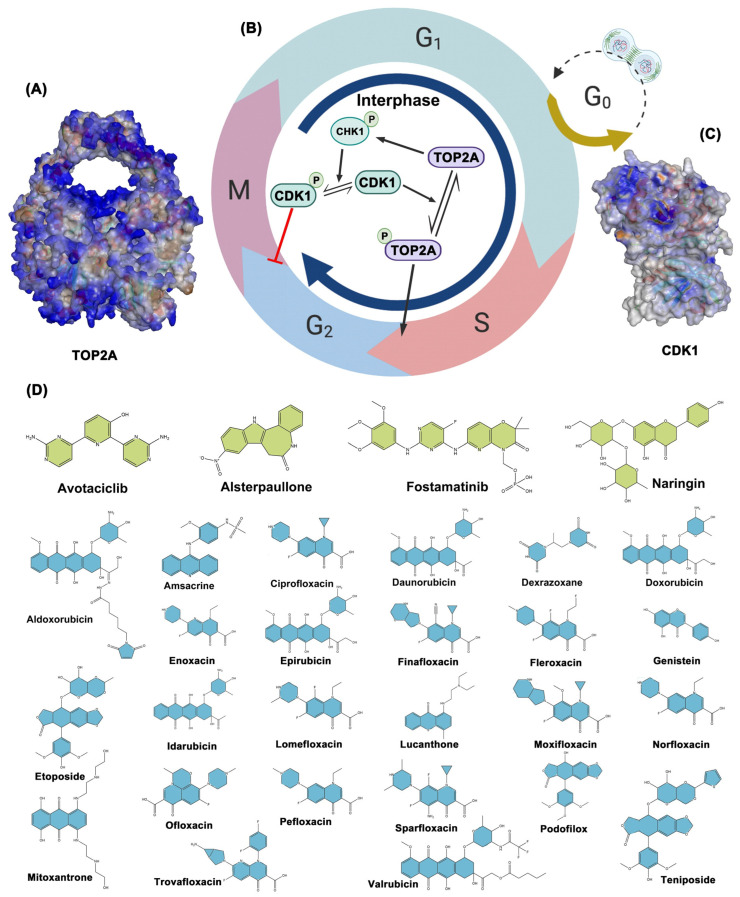
(**A**) Schematic illustration of the 3D structure of the TOP2A protein; (**B**) Schematic representation of the functional interaction network between CDK1 and TOP2A during cell-cycle regulation, (**C**) Schematically illustrates a 3D structure of CDK1 proteins; (**D**) 2D structures of the selected drugs (the green color indicates drugs with CDK1 effects reported, blue drugs with TOP2A effects).

Among the 29 compounds evaluated, 25 FDA-approved or investigational drugs were found to interact with TOP2A by binding at its active site, thereby preventing its productive interaction with DNA and suppressing cancer cell proliferation. Conversely, CDK1 facilitates TOP2A–DNA binding under physiological conditions, rendering its inhibition a therapeutically relevant strategy. Binding of drugs including Amsacrine, Dexrazoxane, Valrubicin, Etoposide, Doxorubicin, Idarubicin, Epirubicin, Podofilox, Genistein, Lomefloxacin, Moxifloxacin, Norfloxacin, Pefloxacin, Sparfloxacin, Trovafloxacin, Ofloxacin, Lucanthone, Daunorubicin, and Aldoxorubicin to the CDK1 protein (PDB ID: 4y72) at the TYR15 phosphorylation site leads to CDK1 phosphorylation, thereby halting cell cycle progression at the G2/M boundary. The consequent reduction or absence of active CDK1 prevents the phosphorylation of TOP2A, impeding its conversion to the phosphorylated isoform and consequently hindering cell cycle advancement from the S phase to the G2 phase. Furthermore, the disruption of TOP2A phosphorylation paradoxically augments total TOP2A levels, which in turn—via CHK1—catalyzes the phosphorylation and inactivation of CDK1, thereby further obstructing G2-to-M phase progression. This dual inhibitory mechanism prevents cancer cell proliferation through two convergent pathways. Additionally, binding of Alsterpaullone, Avotaciclib, Naringin, and Fostamatinib to the TOP2A protein (PDB ID: 6zy5) directly facilitates TOP2A inhibition, culminating in cellular growth arrest. The three-dimensional (3D) structures of the TOP2A and CDK1 proteins are schematically illustrated in Figure 5A and Figure 5C, respectively, while the functional interaction cycles of CDK1 and TOP2A are depicted in Figure 5B. Figure 5B illustrates the proposed regulatory cycle in which phosphorylation-dependent modulation of CDK1 influences TOP2A activity, while CHK1-mediated feedback further contributes to CDK1 inactivation and reinforcement of G2/M cell-cycle arrest. The two-dimensional (2D) chemical structures of all selected compounds are presented in Figure 5D. Of the 29 drugs evaluated in this study, 23 demonstrated multifunctional binding profiles. However, several of these compounds are associated with secondary adverse effects—including nephrotoxicity, hepatotoxicity, and Ames mutagenicity—which necessitate careful clinical consideration in their application.

**Figure 6:**
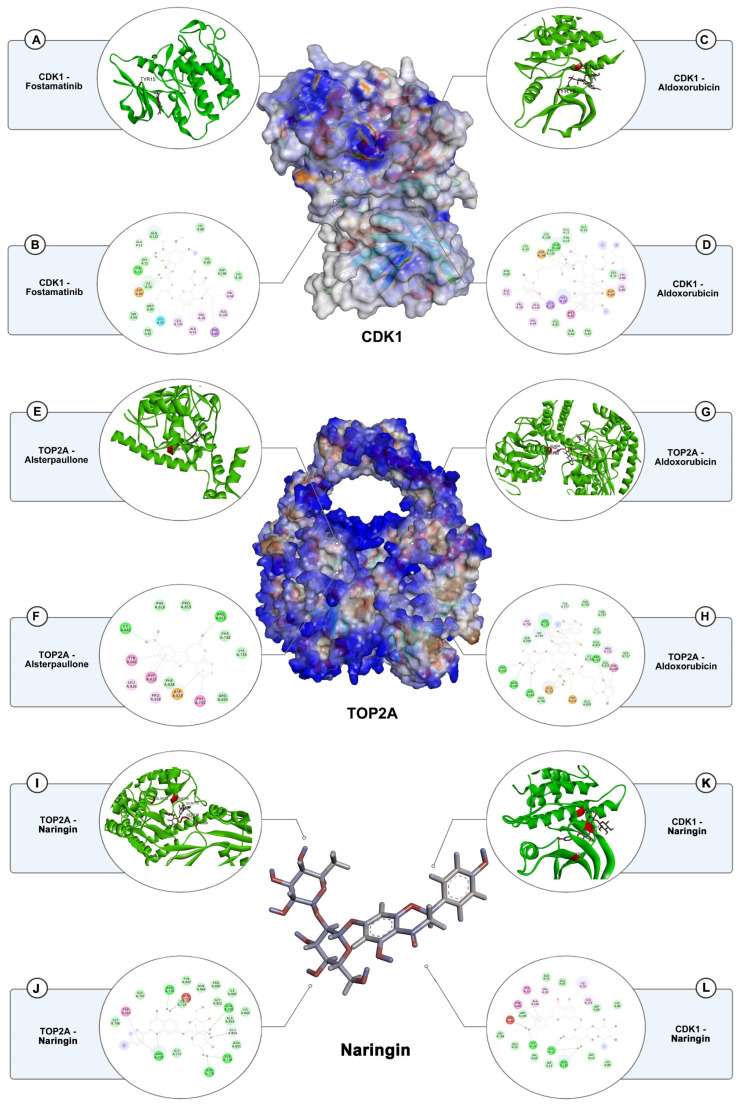
Molecular docking results of selected compounds with CDK1 and TOP2A proteins. (**A**) 3D binding pose of CDK1–Fostamatinib complex; (**B**) 2D interaction diagram of CDK1–Fostamatinib showing atomic bond types; (**C**) 3D binding pose of CDK1–Aldoxorubicin complex; (**D**) 2D interaction diagram of CDK1–Aldoxorubicin; (**E**) 3D binding pose of TOP2A–Alsterpaullone complex; (**F**) 2D interaction diagram of TOP2A–Alsterpaullone; (**G**) 3D binding pose of TOP2A–Aldoxorubicin complex; (**H**) 2D interaction diagram of TOP2A–Aldoxorubicin; (**I**) 3D binding pose of TOP2A–Naringin complex; (**J**) 2D interaction diagram of TOP2A–Naringin; (**K**) 3D binding pose of CDK1–Naringin complex; (**L**) 2D interaction diagram of CDK1–Naringin. Representative high-affinity docking poses are shown for visualization purposes, whereas complete docking results for all 29 compounds against both targets are provided in Supplementary Table S1.

Figure 6 presents the molecular docking results for the two target proteins, CDK1 and TOP2A, with selected high-affinity compounds. For CDK1, the docking interactions with Fostamatinib and Aldoxorubicin are illustrated, representing the compounds with the highest binding affinities among CDK1-targeting drugs (−12.5 and −11.0 kcal/mol, respectively). For TOP2A, the interactions with Alsterpaullone and Aldoxorubicin are displayed, corresponding to the strongest binders identified for this protein (−10.1 and −10.0 kcal/mol, respectively). Comprehensive results of the binding interactions for all 29 drugs with both CDK1 and TOP2A proteins are provided in Supplementary Table S1. The compounds illustrated in Figure 6 were selected solely as representative high-affinity docking complexes for visualization purposes.

#### 2.2.2. Prediction of Medicinal Properties (ADMET)

Table 2 provides comprehensive pharmacokinetic and toxicological insights derived from AdmetSAR database analysis. With respect to subcellular localization, Alsterpaullone, Avotaciclib, Fostamatinib, Naringin, Amsacrine, Valrubicin, Teniposide, Etoposide, Podofilox, Genistein, Fleroxacin, and Aldoxorubicin were predicted to localize within the mitochondria, while Dexrazoxane, Ciprofloxacin, Enoxacin, Lomefloxacin, Moxifloxacin, Norfloxacin, Pefloxacin, Sparfloxacin, Trovafloxacin, Ofloxacin, Lucanthone, and Finafloxacin were predicted to accumulate within lysosomes. Doxorubicin, Idarubicin, Mitoxantrone, Epirubicin, and Daunorubicin were predicted to localize within the nucleus. The AlogP values across all evaluated compounds ranged from −4 to 8.33. The majority of compounds, with the exception of Alsterpaullone, Avotaciclib, Amsacrine, Trovafloxacin, Lucanthone, and Finafloxacin, were predicted not to penetrate the blood–brain barrier. Additionally, 18 drugs—including Alsterpaullone, Amsacrine, Valrubicin, Teniposide, Doxorubicin, Idarubicin, Mitoxantrone, Epirubicin, Ciprofloxacin, Enoxacin, Fleroxacin, Lomefloxacin, Norfloxacin, Pefloxacin, Trovafloxacin, Lucanthone, Daunorubicin, and Aldoxorubicin—were flagged for Ames mutagenicity. Regarding human oral bioavailability, all compounds with the exception of Naringin, Dexrazoxane, Valrubicin, Teniposide, Doxorubicin, Idarubicin, Epirubicin, Podofilox, Genistein, Norfloxacin, Daunorubicin, and Aldoxorubicin demonstrated positive predictions, indicating adequate absorption, acceptable blood-level retention following hepatic metabolism, and renal elimination.

Cross-referencing the docking results from Table 1 with the AdmetSAR data presented in Table 2, the compounds Alsterpaullone, Avotaciclib, Fostamatinib, Naringin, Amsacrine, Dexrazoxane, Valrubicin, Etoposide, Doxorubicin, Idarubicin, Epirubicin, Podofilox, Genistein, Lomefloxacin, Moxifloxacin, Norfloxacin, Pefloxacin, Sparfloxacin, Trovafloxacin, Ofloxacin, Lucanthone, Daunorubicin, and Aldoxorubicin collectively demonstrated significant activity against both CDK1 and TOP2A, facilitating synergistic suppression of cancer cell proliferation. Among these 23 multifunctional compounds, Alsterpaullone and Trovafloxacin exhibited positive AdmetSAR predictions for Ames mutagenicity, renal toxicity, hepatotoxicity, and blood–brain barrier penetration, indicating a non-negligible secondary adverse effect profile that warrants careful clinical consideration. In contrast, Naringin demonstrated favorable predictions across all key safety parameters—including Ames mutagenicity, renal toxicity, hepatotoxicity, and blood–brain barrier penetration—with the notable caveat of a negative prediction for human oral bioavailability, which necessitates careful attention to the route and method of administration to maximize therapeutic efficacy. Collectively, these pharmacokinetic indicators suggest a low risk of secondary drug-induced organ damage, positioning Naringin as a particularly promising candidate for targeted ovarian cancer therapy with a minimal off-target impact on patients undergoing treatment.

#### 2.2.3. Molecular Dynamics Simulations

**Figure 7:**
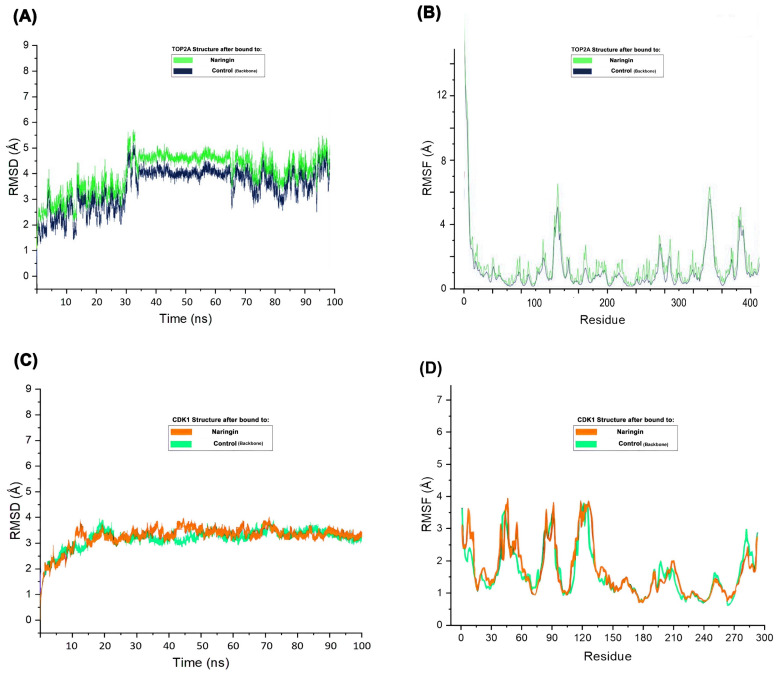
Molecular dynamics simulation of TOP2A and CDK1 in complex with Naringin and Backbone protein. (**A**,**B**) TOP2A (**A**) RMSD (root-mean-square deviation) plot of the protein backbone and ligand within the binding pocket over 100 ns; (**B**) RMSF (root-mean-square fluctuation) plot of residues indicating atomic fluctuations; (**C**,**D**) CDK1 (**C**) RMSD (root-mean-square deviation) plot of the protein backbone and ligand within the binding pocket over 100 ns; (**D**) RMSF (root-mean-square fluctuation) plot of residues indicating atomic fluctuations.

Molecular dynamics simulations were performed to evaluate the structural stability and dynamic behavior of TOP2A and CDK1 in complex with Naringin over a 100 ns trajectory. As illustrated in Figure 7A, the RMSD profile of TOP2A exhibited an initial equilibration phase followed by a relatively stable plateau, indicating that the protein–ligand complex reached conformational stability during the simulation. Notably, the Naringin-bound system showed slightly higher RMSD values compared to the backbone control, suggesting minor conformational adjustments upon ligand binding without compromising overall structural integrity. Consistently, the RMSF analysis (Figure 7B) revealed localized fluctuations predominantly in loop regions, while the core residues remained structurally constrained, indicating that Naringin binding does not induce global destabilization of TOP2A. In the case of CDK1, the RMSD profile (Figure 7C) remained stable throughout the simulation, reflecting a well-maintained binding conformation of Naringin within the active site. Furthermore, RMSF patterns (Figure 7D) demonstrated comparable residue flexibility between the bound and control systems, with only marginal increases in mobility in flexible regions. Collectively, these findings indicate that Naringin forms stable complexes with both TOP2A and CDK1, preserving their global structural frameworks while inducing limited, localized flexibility, which may be functionally relevant for its inhibitory potential.

## 3. Discussion

Tumor initiation and progression are governed by a complex and deeply interconnected constellation of molecular determinants, among which genetic factors and differential gene expression occupy a central position. In essence, malignant growth depends on the coordinated downregulation of tumor-suppressive genes alongside the aberrant upregulation of oncogenic drivers. Comparative microarray analysis of epithelial ovarian cancer cells relative to their normal counterparts revealed substantive differences in transcriptional profiles, and heatmap-based visualization was employed to validate the differential expression patterns identified, ensuring accurate and reproducible group comparisons. In the present investigation, violin plots served a vital analytical role in identifying grouping patterns, inter-sample relationships, and distributional outliers within complex high-dimensional datasets. Three distinct clustering patterns were discerned, with cancer samples exhibiting notably tight data point clustering, suggesting the feasibility of further stratification based on gene expression signatures. The construction of violin plots was carried out with particular methodological care to enable thorough cross-cohort comparisons and to capture the subtleties of data distribution, thereby deepening the analytical robustness of the study.

Subsequent to the transcriptomic analysis, a protein–protein interaction network was constructed to interrogate the functional connectivity among identified gene products. Topological gene parameters—including degree and betweenness centrality—were incorporated into the network to systematically identify and rank protein–protein connections. Following network construction and comparative assessment of gene influence through box plot visualization, *TOP2A* and *CDK1* consistently emerged as the most robustly expressed and topologically central genes within the network, underscoring their pivotal roles in the progression and sustenance of epithelial ovarian cancer. These observations are in concordance with the findings of Zhang et al., who demonstrated through in vitro experimentation that *TOP2A* knockdown suppresses ovarian cancer cell proliferation, induces cell cycle arrest in the G1 phase, and triggers subsequent apoptotic cell death [12]. Complementarily, Qu et al. reported that sinomenine inhibits the proliferation of HeyA8 ovarian cancer cells by attenuating mitotic activity through the downregulation of *CDK1* expression and kinase activity [13].

The enrichment analysis revealed that the hub genes identified in this study converge on several functionally critical pathways, including the p53 signaling pathway, progesterone-mediated oocyte maturation, pyrimidine metabolism, cell cycle regulation, cellular senescence, glutathione metabolism, and oocyte meiosis. In this regard, Schwermer et al. reported that in cells harboring functional p53 signaling, apoptosis constitutes the predominant cellular response to CDK1 inhibition, and that sensitivity to CDK inhibition is determined by p53 status rather than by MYCN expression levels [14]. The precise mechanistic relationship between TOP2A and the p53 signaling pathway, however, remains incompletely characterized [15]. In their investigation, Long et al. demonstrated through phosphorylation array and Western blot analyses that progesterone significantly suppresses AKT and CDK1 phosphorylation, ultimately leading to the activation of p27kip1 within the PI3K/AKT signaling axis [16]. Mochida et al. further established that the initiation of mitosis is orchestrated through the activation of the Cdc2/CDK1 kinase, involving a multi-step regulatory cascade that encompasses cyclin B1 accumulation and nuclear translocation, dephosphorylation of Cdc2/CDK1 at Thr14 and Tyr15, and phosphorylation at Thr161 [17].

Interpretation of the Spearman correlation coefficient requires consideration of both its magnitude and directionality. Coefficients approaching 1 or −1 signify strong monotonic associations, while values proximate to 0 reflect weak or absent correlations. A positive coefficient indicates a direct relationship, whereas a negative coefficient denotes an inverse association. The Spearman correlation coefficient of 0.71 observed between *CDK1* and *TOP2A* confirms a statistically significant and functionally meaningful positive co-expression relationship between these genes, whereby increased expression of one gene is proportionally associated with increased expression of the other. Nonetheless, the difference in correlation strength between the normal and tumor groups—as reflected by their respective Pearson R-values of 0.84 and 0.42—implies that the nature of the *CDK1*–*TOP2A* relationship may be modulated by biological context. This variation may reflect the influence of additional genetic alterations, epigenetic modifications, or microenvironmental factors operative within the tumor setting. These observations are consistent with the findings of Li et al., who demonstrated that elevated expression of CDK1 (HR 1.27, 95% CI 1.11–1.46, *p* = 6 × 10^−4^) and *TOP2A* (HR 1.27, 95% CI 1.11–1.44, *p* = 3.9 × 10^−4^) has been reported in previous independent cohorts to exhibit associations with unfavorable clinical outcomes in epithelial ovarian cancer [18]. Furthermore, multiple independent studies have confirmed that *CDK1* expression levels modulate the aggressiveness of diverse malignancies—including lung, colorectal, breast, and ovarian cancers—by regulating key proliferative signaling pathways [19,20].

As the most prominently upregulated genes in ovarian cancer, *TOP2A* and *CDK1* have been demonstrated to play indispensable roles in cell cycle progression. *CDK1* contributes to G2/M transition regulation through phosphorylation-dependent signaling events, while *TOP2A* participates in DNA replication and chromosome segregation, collectively supporting a coordinated cell-cycle regulatory network relevant to ovarian cancer progression. The functional pathway of *TOP2A* is such that its phosphorylation is prerequisite for continuation of the cell cycle, enabling the successive and uninterrupted completion of the mitotic process. As illustrated in Figure 5B, CDK1 is a necessary upstream effector for the phosphorylation of TOP2A. Pharmacological targeting of *TOP2A* with appropriate inhibitory compounds can therefore suppress its phosphorylation, arresting the cell cycle at the S/G2 boundary. Moreover, through its interaction with *CHK1*, *TOP2A* promotes *CDK1* phosphorylation, thereby inhibiting cell cycle advancement from the G2 phase to the M phase. Reciprocally, *CDK1* phosphorylation can attenuate the *TOP2A* phosphorylation pathway, further decelerating cell cycle kinetics. Independent inhibition of *CDK1* through its phosphorylation at TYR15 is likewise of considerable therapeutic significance, as it directly induces G2/M phase arrest. These observations align with those of Khedkar et al., who highlighted the significance of a complex feedback signaling network in facilitating meiotic maturation and ensuring successful oocyte meiotic progression, while also reporting that the upregulation of *TTK*, *NEK2*, and *CDK1* is linked to growth factor-induced signaling cascades and that deregulation of salvage pyrimidine and purine pathways—common in cancer—promotes elevated anabolic metabolism conducive to macromolecular biosynthesis [21]. As a pivotal nuclear protein, *TOP2A* inhibition has been shown to suppress invasion, metastasis, and proliferation; furthermore, through *CHK1* phosphorylation, *TOP2A* impedes G2-to-M phase progression and facilitates the epithelial-to-mesenchymal transition (EMT) [22]. TOP2A is essential for DNA replication and cell division and is characteristically overexpressed in proliferating cells [22,23], with established associations across multiple human malignancies including ovarian [12], colon [24], breast, nasopharyngeal, and renal cell carcinomas [21]. In light of these properties, multiple classes of *TOP2A* inhibitors have demonstrated promise as anticancer therapeutic agents [25].

Several investigations have established that CDK1 phosphorylation at the Tyr15 site restricts its kinase activity, thereby impeding G2 phase cell proliferation [26,27]. As serine/threonine kinases, cyclin-dependent kinases are essential coordinators of cell cycle progression in eukaryotes [28], with three of the thirteen known CDK family members playing direct regulatory roles in cell cycle control [29]. The G2/M transition in eukaryotes is primarily governed by the CDK1/Cyclin B1 complex, and attenuation of CDK1 activity effectively obstructs this critical transition point [30]. Notably, cisplatin-resistant ovarian cancer cells have been reported to harbor elevated CDK1 expression, implicating this kinase in the molecular basis of platinum resistance [31]. The robust mechanistic and co-expression linkage between TOP2A and CDK1 in ovarian cancer, corroborated through pathway analyses and cross-validation with resources including GEPIA and Oncomine, confirms their mutual regulatory interdependence, with the overexpression of both genes collectively facilitating ovarian cancer development and progression.

Given the extensive inhibitory binding sites of TOP2A, this protein has proven receptive to a diverse range of pharmacological inhibitors and has demonstrated promising responses to the majority of these treatments [25]. As a central regulator of the entire cell cycle, CDK1 has similarly emerged as a compelling and viable target for cancer therapeutics [8]. Prior investigations have documented the CDK1-inhibitory activities of compounds including Fostamatinib [32], Alsterpaullone [33], Seliciclib [34], and Olomoucine, while drugs such as Dexrazoxane, Teniposide [35], Doxorubicin [36], Etoposide [37], Idarubicin [38], Mitoxantrone [39], Epirubicin [40], Podofilox [41], Genistein [42], Ciprofloxacin [43], Pefloxacin [44], Lucanthone [45], and Daunorubicin [46] have been established as TOP2A-targeting agents with demonstrable anticancer activity. Naringin has additionally been documented to inhibit ovarian cancer cell growth [21,47]. In the current study, in silico and molecular docking analyses confirmed the inhibitory effects of all 29 evaluated compounds on TOP2A. Furthermore, the capacity of Alsterpaullone, Avotaciclib, Fostamatinib, Naringin, Amsacrine, Dexrazoxane, Valrubicin, Etoposide, Doxorubicin, Idarubicin, Epirubicin, Podofilox, Genistein, Lomefloxacin, Moxifloxacin, Norfloxacin, Pefloxacin, Sparfloxacin, Trovafloxacin, Ofloxacin, Lucanthone, Daunorubicin, and Aldoxorubicin to induce CDK1 phosphorylation—thereby augmenting TOP2A inhibition through prevention of TOP2A phosphorylation—was computationally validated. These computational observations suggest the potential relevance of dual-target modulation of CDK1 and TOP2A in epithelial ovarian cancer and warrant further experimental investigation.

AdmetSAR-based pharmacodynamic profiling was employed to predict the safety and pharmacokinetic attributes of the evaluated compounds, enabling the identification of agents with favorable efficacy-to-toxicity ratios [48,49]. Among the 23 multifunctional compounds demonstrating activity against both CDK1 and TOP2A, Alsterpaullone and Trovafloxacin exhibited strong binding affinities (Alsterpaullone–CDK1: −10.9 kcal/mol; Alsterpaullone–TOP2A: −10.1 kcal/mol; Trovafloxacin–CDK1: −9.9 kcal/mol; Trovafloxacin–TOP2A: −8.5 kcal/mol); however, both compounds were associated with nephrotoxicity, hepatotoxicity, Ames mutagenicity, and blood–brain barrier penetration, necessitating careful prescribing consideration. In contrast, Naringin did not induce nephrotoxicity, hepatotoxicity, or Ames mutagenicity, nor did it demonstrate blood–brain barrier penetration, thereby imposing a substantially lower burden of secondary drug-related injury on patients undergoing treatment, and supporting its prioritization as a computational candidate for subsequent experimental evaluation. Accordingly, Naringin was prioritized for subsequent computational analyses based on its favorable dual-target binding profile, acceptable ADMET characteristics, low predicted toxicity, and stable interaction behavior across both protein complexes. Molecular dynamics simulations further supported these findings by demonstrating stable binding of Naringin to both TOP2A and CDK1, with limited structural deviations and only localized residue fluctuations, confirming the robustness of the predicted protein–ligand interactions under dynamic conditions [4]. Despite the integration of multiple independent transcriptomic datasets and computational validation platforms, additional experimental studies including qRT-PCR, Western blotting, and immunohistochemical analyses in EOC models are warranted to further validate the biological and translational relevance of the identified targets.

Several limitations should be considered when interpreting the present findings. This study was primarily computational and lacked experimental validation in cellular or clinical epithelial ovarian cancer models. In addition, molecular docking and molecular dynamics simulations provide predictive rather than definitive evidence of therapeutic efficacy or mechanistic inhibition. Future experimental investigations are therefore required to validate the biological, pharmacological, and translational relevance of the identified targets and candidate compounds.

## 4. Materials and Methods

### 4.1. Handling Extensive Raw Data

Raw Cell Expression (CEL) files were retrieved from the NCBI Gene Expression Omnibus (GEO) database (https://www.ncbi.nlm.nih.gov/geo/, accessed on 21 March 2026) [50]. The gene expression dataset for epithelial ovarian cancer was obtained from GEO accession GSE28799, while the corresponding data for normal ovarian epithelial cells were acquired from GSE54388 and GSE14407. Data normalization and quality filtering were performed using the Transcriptome Analysis Console (TAC) software 4.0.1 (Thermo Fisher Scientific, Waltham, MA, USA; https://www.thermofisher.com, accessed on 21 March 2026), yielding a refined set of 21 overlapping differentially expressed genes for downstream analysis. The R 4.3.2 programming language was employed to quantitatively assess gene expression levels across the defined study groups [51]. To minimize potential dataset-specific technical variation, normalization consistency and sample distribution patterns were additionally evaluated through principal component analysis prior to downstream DEG analyses. A stringent Log2FC threshold was intentionally applied to prioritize highly robust and consistently dysregulated genes across independent datasets while minimizing false-positive findings. Additional exploratory analyses using lower expression thresholds demonstrated persistent identification of *CDK1* and *TOP2A* among the dominant hub genes. Principal Component Analysis (PCA) and heatmap visualization were subsequently generated to characterize and compare the molecular profiles of the study groups.

### 4.2. Protein Interaction Network

Given the functional relevance of gene connectivity in network-level analyses, the top 100 upregulated genes ranked by differential expression were initially utilized for exploratory protein–protein interaction network construction, whereas the final downstream analyses and hub-gene prioritization were subsequently refined to the 21 overlapping highly stringent differentially expressed genes. These genes were queried within the STRING online database (https://string-db.org, accessed on 21 March 2026) to determine their pairwise interaction associations [52]. The resulting protein–protein interaction network was visualized using Cytoscape software 3.10.0 (https://cytoscape.org/, accessed on 21 March 2026), with gene topological parameters incorporated for comprehensive network analysis and comparative evaluation [53]. Results were represented as box plots to facilitate clear and quantitative assessment of relative gene strengths within the network.

### 4.3. Validation and Correlation of Genes

The expression profiles of *CDK1* and *TOP2A* were examined across a broad panel of human tumor types, and pan-cancer box plots were generated to characterize their differential expression. Gene-level validation was independently performed using the TNMplot online database (https://tnmplot.com/analysis/, accessed on 21 March 2026) [54], with separate expression plots generated for normal, tumor, and metastatic tissue groups. The strength and directionality of the co-expression relationship between *CDK1* and *TOP2A* were evaluated using both Spearman and Pearson correlation methodologies. The Spearman rank correlation coefficient was employed to assess the monotonic relationship between the two variables, with results visualized in a correlation heatmap, while the Pearson correlation coefficient was used to quantify the linear relationship between the continuous expression variables, depicted using scatter plots. Gene expression profiling and survival analyses for *CDK1* and *TOP2A* were conducted using the Gene Expression Profiling Interactive Analysis 2 (GEPIA2) platform (http://gepia2.cancer-pku.cn/, accessed on 21 March 2026), which integrates RNA-seq data from the TCGA and GTEx databases (https://xenabrowser.net/datapages/, accessed on 21 March 2026). Boxplots were generated to compare gene expression levels between ovarian cancer and matched normal tissues, with expression values presented as log2-transformed transcripts per million (TPM). Statistical significance between groups was evaluated using the Mann–Whitney U test as implemented within GEPIA2. Overall survival analyses were conducted using the Kaplan–Meier method, with patients stratified into high- and low-expression groups based on the median expression level of each respective gene. Inter-group survival differences were assessed using the log-rank test, and hazard ratios (HRs) with corresponding confidence estimates were automatically calculated by the GEPIA2 platform. All parameters were analyzed using default GEPIA2 settings unless otherwise specified.

### 4.4. Pharmacological Effects In Silico

Through systematic examination of hub gene expression within the context of gene interaction networks and functional pathway analyses, TOP2A and CDK1 were identified as high-priority targets for pharmacological intervention aimed at controlling tumor growth. The selected 29 compounds were curated through literature-supported screening based on previously reported anticancer activity, documented interactions with cell-cycle regulatory pathways, and known or predicted relevance to CDK1- or TOP2A-associated molecular mechanisms. The protein structures of human TOP2A and CDK1 were retrieved from the UniProt database (https://www.uniprot.org/, accessed on 27 March 2026) and subsequently extracted from the RCSB Protein Data Bank (RCSB PDB, https://www.rcsb.org/, (accessed on 27 March 2026)) [55]. Protein preparation was performed using the UCSF Chimera program 1.17.2 (University of California, San Francisco, CA, USA), encompassing the removal of co-crystallized ligands and extraneous water molecules, addition of hydrogen atoms, elimination of non-essential residues, amino acid charge assignment, and energy minimization of the protein structure [56,57], thereby yielding structures suitable for high-quality molecular docking. Pharmacologically relevant binding sites were identified and extracted from the COACH database (https://zhanggroup.org/COACH/, accessed on 27 March 2026) [58] and the literature section of the PDB database for use in the final docking protocol.

The two-dimensional (2D) chemical structures of the 29 candidate compounds were retrieved from the PubChem database (https://pubchem.ncbi.nlm.nih.gov/, accessed on 27 March 2026) and assigned using IUPAC nomenclature. Three-dimensional (3D) structural conversions were performed using ChemBio3D software 12.0 (PerkinElmer Informatics, Inc., Waltham, MA, USA), with energy minimization and molecular dynamics simulations applied to achieve the most stable and physiologically relevant molecular conformations [59]. Molecular docking was orchestrated using AutoDock Vina 1.2.x (The Scripps Research Institute, La Jolla, CA, USA) [60,61] in conjunction with PyRX 1.0 (The Scripps Research Institute, La Jolla, CA, USA) [62], enabling precise identification of ligand binding poses within the target protein active sites. Pharmacokinetic and toxicological attributes of the selected compounds were subsequently evaluated using the Protein–Ligand Interaction Profiler (PLIP, https://plip-tool.biotec.tu-dresden.de/plip-web/plip/index, accessed on 27 March 2026) [63] and the AdmetSAR 3 online platform (https://lmmd.ecust.edu.cn/admetsar3, accessed on 27 March 2026) [49].

Molecular dynamics (MD) simulations were conducted to investigate the stability and conformational dynamics of TOP2A and CDK1 in both apo (backbone) and Naringin-bound states using GROMACS 2023.3 (GROMACS development teams at the Royal Institute of Technology and Uppsala University, Stockholm/Uppsala, Sweden). The CHARMM36 force field was employed for protein parameterization, while ligand topology parameters were generated using a compatible force field approach. Each system was solvated in a cubic box using the TIP3P water model, and appropriate counter-ions were added to neutralize the system. Energy minimization was performed using the steepest descent algorithm to eliminate steric clashes, followed by equilibration under constant volume (NVT) and constant pressure (NPT) ensembles to stabilize temperature and pressure conditions. Subsequently, a 100 ns production run was carried out under periodic boundary conditions. Long-range electrostatic interactions were treated using the Particle Mesh Ewald (PME) method, and bond constraints were applied using the LINCS algorithm. Trajectory analyses were performed to calculate root-mean-square deviation (RMSD) and root-mean-square fluctuation (RMSF) to assess structural stability and residue-level flexibility. These simulations were conducted for both target proteins under identical conditions to ensure consistency and enable reliable analysis of ligand-induced effects.

### 4.5. Statistical Analysis

Quantitative analyses were carried out within a dual-platform computational environment comprising R (version 4.3.2; R Core Team, Vienna, Austria) and Python 3.11. Differential expression profiling was conducted through the limma framework 3.21, wherein the Benjamini–Hochberg correction was systematically applied to account for multiple comparisons and maintain stringent false discovery rate control [64]. Topological characterization of the constructed gene interaction networks was accomplished via igraph 2.1.4 (igraph Development Team, Vienna, Austria), while functional enrichment analyses were executed using clusterProfiler 3.21 (Bioconductor project, Guangzhou Medical University, Guangzhou, China). Survival association modeling was performed through the dedicated survival package, and all resulting data were rendered into publication-quality visualizations using ggplot2 in R 4.3.2 and matplotlib 3.9. Principal component analysis was performed using the factoextra package 1.0.7, while clustered heatmap visualization was generated using the pheatmap package 1.0.12 within the R 4.3.2 environment. A significance threshold of *p* < 0.05 was adopted uniformly across all analyses, with FDR-adjusted *p*-values additionally reported for multiplicity-corrected comparisons [65,66].

## 5. Conclusions

This study employed an integrated transcriptomic and computational framework to investigate the molecular underpinnings of epithelial ovarian cancer, identifying *CDK1* and *TOP2A* as consistently overexpressed and topologically central regulators of cell-cycle progression across independent transcriptomic datasets. The principal novelty of this work resides in the systematic dual-target pharmacological evaluation of these two functionally interconnected proteins within a unified EOC-specific pipeline, extending beyond conventional single-target paradigms. The convergence of independent transcriptomic, network-based, and computational pharmacology approaches within a single analytical framework strengthens the robustness of the reported observations. Through coordinated network topology analysis, functional enrichment, molecular docking, ADMET profiling, and molecular dynamics simulations, Naringin was prioritized as a preliminary dual-target candidate exhibiting favorable binding characteristics, conformational stability, and an acceptable predicted safety profile. Nevertheless, as the findings remain computational in nature, experimental validation in relevant cellular and preclinical models constitutes an essential next step toward confirming their biological and translational relevance.

## Figures and Tables

**Table 1:** The results of AutoDock Vina (binding affinity) and PLIP database.

	Prediction	CDK1 (4y72)				TOP2A (6zy5)			
Drug		Binding Affinity (kcal/mol)	Hydrophobic Interactions	Hydrogen Bonds	Salt Bridges	Binding Affinity (kcal/mol)	Hydrophobic Interactions	Hydrogen Bonds	Salt Bridges
Alsterpaullone		−10.9	8	*	*	−10.1	10	3	1
Avotaciclib		−9.3	1	2	*	−8.3	1	1	*
Fostamatinib		−12.5	4	2	*	−9	1	4	1
Naringin		−10.6	6	8	*	−9.4	1	11	1
Amsacrine		−10.8	8	1	*	−9.9	6	6	*
Dexrazoxane		−6.8	*	2	1	−6.6	*	6	1
Valrubicin		−9.8	8	4	*	−9.5	3	5	1
Teniposide		*	*	*	*	−9.5	*	2	1
Etoposide		−7.5	4	1	*	−9.6	3	7	2
Doxorubicin		−10.2	5	5	*	−8.7	3	5	*
Idarubicin		−10.5	7	4	*	−9.7	6	5	1
Mitoxantrone		*	*	*	*	−7.5	*	10	*
Epirubicin		−10.3	6	4	*	−8.7	3	6	*
Podofilox		−10.4	6	4	*	−7.6	5	6	*
Genistein		−9.2	7	7	*	−9.3	5	6	*
Ciprofloxacin		*	*	*	*	−7.7	2	4	2
Enoxacin		*	*	*	*	−8.8	3	4	2
Fleroxacin		*	*	*	*	−9.1	5	4	*
Lomefloxacin		−9.4	4	3	2	−8	1	6	*
Moxifloxacin		−6.3	2	*	*	−8.2	2	3	*
Norfloxacin		−9.2	3	3	*	−7.6	4	3	2
Pefloxacin		−7.7	5	3	*	−9.4	7	4	*
Sparfloxacin		−8.3	4	4	2	−7.6	3	6	1
Trovafloxacin		−9.9	5	1	*	−8.5	3	2	2
Ofloxacin		−9.2	4	3	1	−7.2	1	3	*
Lucanthone		−8.5	6	1	*	−6.8	*	1	1
Daunorubicin		−10.6	5	4	*	−9.6	3	6	*
Finafloxacin		*	*	*	*	−8.3	1	3	1
Aldoxorubicin		−11	13	2	1	−10	5	8	3

* The asterisk indicates a lack of binding at the identified binding site in the target protein.

**Table 2:** The results of AdmetSAR database.

Name of the Drug	Admet SAR							
	Subcellular Localization	AlogP	Molecular Weight	Blood–Brain Barrier	Human Oral Bioavailability	Nephrotoxicity	Hepatotoxicity	Ames Mutagenesis
Alsterpaullone	Mitochondria	3.24	293.276	+	0.7143 (+)	0.5739 (+)	0.7875 (+)	0.88 (+)
Avotaciclib	Mitochondria	0.87	281.279	+	0.5571 (+)	0.4864 (+)	0.6125 (+)	0.5 (−)
Fostamatinib	Mitochondria	3.09	580.459	−	0.5571 (+)	0.7326 (+)	0.6677 (+)	0.53 (−)
Naringin	Mitochondria	−1.17	580.54	−	0.9857 (−)	0.6977 (−)	0.8750 (−)	0.61 (−)
Amsacrine	Mitochondria	4.51	393.459	+	0.7 (+)	0.5909 (−)	0.7 (+)	0.95 (+)
Dexrazoxane	Lysosomes	−2.71	268.269	−	0.6857 (−)	0.5807 (+)	0.875 (+)	0.88 (−)
Valrubicin	Mitochondria	2.64	723.651	−	0.9 (−)	0.5 (+)	0.825 (−)	0.7863 (+)
Teniposide	Mitochondria	2.75	656.654	−	0.7714 (−)	0.6168 (+)	0.9 (+)	0.64 (+)
Etoposide	Mitochondria	1.34	588.556	−	0.6286 (+)	0.7837 (+)	0.8125 (+)	0.81 (−)
Doxorubicin	Nucleus	0	543.519	−	0.9143 (−)	0.5 (+)	0.85 (−)	0.99 (+)
Idarubicin	Nucleus	1.02	497.493	−	0.9429 (−)	0.6078 (−)	0.875 (−)	1 (+)
Mitoxantrone	Nucleus	−0.14	444.480	−	0.5143 (+)	0.7839 (+)	0.7785 (+)	0.9 (+)
Epirubicin	Nucleus	0	543.519	−	0.9143 (−)	0.5 (+)	0.85 (−)	0.99 (+)
Podofilox	Mitochondria	2.41	414.405	−	0.5143 (−)	0.7839 (+)	0.5375 (+)	0.82 (−)
Genistein	Mitochondria	2.58	270.236	−	0.6 (−)	0.6869 (+)	0.6179 (+)	0.63 (−)
Ciprofloxacin	Lysosomes	1.58	331.341	−	0.7143 (+)	0.7839 (+)	0.975 (+)	0.8072 (+)
Enoxacin	Lysosomes	0.66	320.318	−	0.8 (+)	0.5588 (+)	0.9375 (+)	0.93 (+)
Fleroxacin	Mitochondria	1.7	369.344	−	0.9857 (+)	0.798 (−)	0.825 (+)	0.896 (+)
Lomefloxacin	Lysosomes	1.8	351.347	−	0.9857 (+)	0.8077 (−)	0.75 (−)	0.89 (+)
Moxifloxacin	Lysosomes	2.37	401.431	−	0.9429 (+)	0.6406 (−)	0.975 (+)	0.5382 (−)
Norfloxacin	Lysosomes	1.27	319.330	−	0.5714 (−)	0.5974 (+)	0.9125 (+)	0.86 (+)
Pefloxacin	Lysosomes	1.61	333.357	−	0.8571 (+)	0.6217 (−)	0.9625 (+)	0.9 (+)
Sparfloxacin	Lysosomes	2.08	392.399	−	0.9571 (+)	0.7608 (+)	0.975 (+)	0.55 (−)
Trovafloxacin	Lysosomes	1.89	416.36	+	0.9143 (+)	0.6095 (+)	0.9428 (+)	0.65 (+)
Ofloxacin	Lysosomes	1.54	361.367	−	1 (+)	0.8032 (−)	0.975 (+)	0.5677 (−)
Lucanthone	Lysosomes	4.48	340.482	+	0.7714 (+)	0.7894 (−)	0.825 (+)	0.94 (+)
Daunorubicin	Nucleus	1.03	527.519	−	0.9143 (−)	0.5573 (+)	0.95 (−)	1 (+)
Finafloxacin	Lysosomes	1.22	398.394	+	0.8714 (+)	0.5285 (+)	0.8625 (+)	0.5432 (−)
Aldoxorubicin	Mitochondria	0.39	750.758	−	0.8286 (−)	0.7199 (−)	0.7613 (−)	0.74 (+)

## Data Availability

The datasets generated and/or analyzed during the current study are available in the [GSE DataSets] repository, [https://www.ncbi.nlm.nih.gov/geo/query/acc.cgi?acc=GSE28799, (accessed on 21 March 2026) and https://www.ncbi.nlm.nih.gov/geo/query/acc.cgi?acc=GSE54388, (accessed on 21 March 2026)].

## Supplementary Materials

The following supporting information can be downloaded at: https://www.mdpi.com/article/10.3390/ijms27125264/s1.

## Abbreviations

The following abbreviations are used in this manuscript:

CDK1	Cyclin-dependent kinase 1
TOP2A	DNA Topoisomerase II Alpha
EOC	Epithelial ovarian cancer
PPI	Protein–Protein Interaction
PCA	Principal Component Analysis
RCSB PDB	RCSB Protein Data Bank
PLIP	Protein–Ligand Interaction Profiler
DEGs	Differentially Expressed Genes
MD	Molecular dynamics
BCL2	B-cell leukemia/lymphoma 2 protein
ESR1	Estrogen receptor 1
CHEK1	Checkpoint kinase 1
KPNA2	Karyopherin α2
GO	Gene ontology
KIF11	Kinesin-like protein KIF11
CENPE	Centromere-associated protein E
NUSAP1	Nucleolar and spindle-associated protein 1

## References

[1] Li S., Yang S., Hong Y. (2022). Higher thymocyte selection-associated high mobility group box (TOX) expression predicts poor prognosis in patients with ovarian cancer. BMC Cancer.

[2] Bernstock J.D., Gary S.E., Klinger N., Valdes P.A., Ibn Essayed W., Olsen H.E., Chagoya G., Elsayed G., Yamashita D., Schuss P. (2022). Standard clinical approaches and emerging modalities for glioblastoma imaging. Neuro-Oncol. Adv..

[3] Wang E.W., Wei C.H., Liu S., Lee S.J.-J., Shehayeb S., Glaser S., Li R., Saadat S., Shen J., Dellinger T. (2020). Frontline Management of Epithelial Ovarian Cancer—Combining Clinical Expertise with Community Practice Collaboration and Cutting-Edge Research. J. Clin. Med..

[4] Masoudi M., Samadiafshar S., Azizi H., Skutella T. (2025). Integrated Analysis, Machine Learning, Molecular Docking and Dynamics of CDK1 Inhibitors in Epithelial Ovarian Cancer: A Multifaceted Approach Towards Targeted Therapy. Int. J. Mol. Sci..

[5] Liu C.-L., Yuan R.-H., Mao T.-L. (2021). The molecular landscape influencing prognoses of epithelial ovarian cancer. Biomolecules.

[6] Sowamber R., Lukey A., Huntsman D., Hanley G. (2023). Ovarian cancer: From precursor lesion identification to population-based prevention programs. Curr. Oncol..

[7] Craig O., Nigam A., Dall G.V., Gorringe K. (2023). Rare epithelial ovarian cancers: Low grade serous and mucinous carcinomas. Cold Spring Harb. Perspect. Med..

[8] Fu L., Mou J., Deng Y., Ren X., Qiu S. (2022). Design, synthesis, and activity assays of cyclin-dependent kinase 1 inhibitors with flavone scaffolds. Front. Chem..

[9] Jafari A., Babajani A., Forooshani R.S., Yazdani M., Rezaei-Tavirani M. (2022). Clinical applications and anticancer effects of antimicrobial peptides: From bench to bedside. Front. Oncol..

[10] Celis S., Hobor F., James T., Bartlett G.J., Ibarra A.A., Shoemark D.K., Hegedüs Z., Hetherington K., Woolfson D.N., Sessions R.B. (2021). Query-guided protein–protein interaction inhibitor discovery. Chem. Sci..

[11] Li Y.-L., Mao J., Cheng Z., Zhou X.-Y., Zhang D.-N., Li Y.-Z., Cao Z.-X., Ren J.-X. (2026). Identification of low-toxicity DNA topoisomerase I inhibitors with potential blood–brain barrier penetrability for glioblastoma therapy: Structure-based virtual screening reveals promising novel Scaffolds. Mol. Divers..

[12] Zhang K., Zheng X., Sun Y., Feng X., Wu X., Liu W., Gao C., Yan Y., Tian W., Wang Y. (2024). TOP2A modulates signaling via the AKT/mTOR pathway to promote ovarian cancer cell proliferation. Cancer Biol. Ther..

[13] Qu X., Yu B., Zhu M., Li X., Ma L., Liu C., Zhang Y., Cheng Z. (2021). Sinomenine inhibits the growth of ovarian cancer cells through the suppression of mitosis by down-regulating the expression and the activity of CDK1. OncoTargets Ther..

[14] Schwermer M., Lee S., Köster J., van Maerken T., Stephan H., Eggert A., Morik K., Schulte J.H., Schramm A. (2015). Sensitivity to cdk1-inhibition is modulated by p53 status in preclinical models of embryonal tumors. Oncotarget.

[15] Kou F., Sun H., Wu L., Li B., Zhang B., Wang X., Yang L. (2020). TOP2A promotes lung adenocarcinoma cells’ malignant progression and predicts poor prognosis in lung adenocarcinoma. J. Cancer.

[16] Long H., Yu W., Yu S., Yin M., Wu L., Chen Q., Cai R., Suo L., Wang L., Lyu Q. (2021). Progesterone affects clinic oocyte yields by coordinating with follicle stimulating hormone via PI3K/AKT and MAPK pathways. J. Adv. Res..

[17] Mochida S., Rata S., Hino H., Nagai T., Novák B. (2016). Two bistable switches govern M phase entry. Curr. Biol..

[18] Li W., Liu Z., Liang B., Chen S., Zhang X., Tong X., Lou W., Le L., Tang X., Fu F. (2018). Identification of core genes in ovarian cancer by an integrative meta-analysis. J. Ovarian Res..

[19] Izadi S., Nikkhoo A., Hojjat-Farsangi M., Namdar A., Azizi G., Mohammadi H., Yousefi M., Jadidi-Niaragh F. (2020). CDK1 in breast cancer: Implications for theranostic potential. Anti-Cancer Agents Med. Chem..

[20] Li M., He F., Zhang Z., Xiang Z., Hu D. (2020). CDK1 serves as a potential prognostic biomarker and target for lung cancer. J. Int. Med. Res..

[21] Khedkar H.N., Wang Y.-C., Yadav V.K., Srivastava P., Lawal B., Mokgautsi N., Sumitra M.R., Wu A.T.H., Huang H.-S. (2021). In-silico evaluation of genetic alterations in ovarian carcinoma and therapeutic efficacy of NSC777201, as a novel multi-target agent for TTK, NEK2, and CDK1. Int. J. Mol. Sci..

[22] Wang T., Lu J., Wang R., Cao W., Xu J. (2022). TOP2A promotes proliferation and metastasis of hepatocellular carcinoma regulated by miR-144-3p. J. Cancer.

[23] Grammatikaki S., Bala V.-M., Katifelis H., Lampropoulou D.I., Mukha I., Vityuk N., Lagopati N., Kouloulias V., Aravantinos G., Gazouli M. (2024). Fe_3_O_4_ and Fe_3_O_4core_ Au_shell_-Based Hyperthermia Reduces Expression of Proliferation Markers *Ki-67, TOP2A* and *TPX2* in a Human Breast Cancer Cell Line. In Vivo.

[24] Li H., Wang P., Chen H., Shao Y., Luo H. (2025). NEIL3 and TOP2A as key drivers of esophageal cancer through WNT signaling. Biomol. Biomed..

[25] Mehraj U., Qayoom H., Shafi S., Farhana P., Asdaq S.M.B., Mir M.A. (2022). Cryptolepine targets TOP2A and inhibits tumor cell proliferation in breast cancer cells—An in vitro and in silico study. Anti-Cancer Agents Med. Chem..

[26] Zhang R., Shi H., Ren F., Zhang M., Ji P., Wang W., Liu C. (2017). The aberrant upstream pathway regulations of CDK1 protein were implicated in the proliferation and apoptosis of ovarian cancer cells. J. Ovarian Res..

[27] Cedeno-Rosario L., Honda D., Sunderland A.M., Lewandowski M.D., Taylor W.R., Chadee D.N. (2022). Phosphorylation of mixed lineage kinase MLK3 by cyclin-dependent kinases CDK1 and CDK2 controls ovarian cancer cell division. J. Biol. Chem..

[28] Chohan T.A., Qayyum A., Rehman K., Tariq M., Akash M.S.H. (2018). An insight into the emerging role of cyclin-dependent kinase inhibitors as potential therapeutic agents for the treatment of advanced cancers. Biomed. Pharmacother..

[29] Deota S., Rathnachalam S., Namrata K., Boob M., Fulzele A., Radhika S., Ganguli S., Balaji C., Kaypee S., Vishwakarma K.K. (2019). Allosteric regulation of cyclin-B binding by the charge state of catalytic lysine in CDK1 is essential for cell-cycle progression. J. Mol. Biol..

[30] Roskoski R. (2019). Cyclin-dependent protein serine/threonine kinase inhibitors as anticancer drugs. Pharmacol. Res..

[31] Li J., Zhi X., Shen X., Chen C., Yuan L., Dong X., Zhu C., Yao L., Chen M. (2020). Depletion of UBE2C reduces ovarian cancer malignancy and reverses cisplatin resistance via downregulating CDK1. Biochem. Biophys. Res. Commun..

[32] Senturk A., Sahin A.T., Armutlu A., Kiremit M.C., Acar O., Erdem S., Bagbudar S., Esen T., Ozlu N. (2022). Quantitative Phosphoproteomics Analysis Uncovers PAK2- and CDK1-Mediated Malignant Signaling Pathways in Clear Cell Renal Cell Carcinoma. Mol. Cell. Proteom..

[33] Soni D.V., Jacobberger J.W. (2004). Inhibition of cdk1 by alsterpaullone and thioflavopiridol correlates with increased transit time from mid G2 through prophase. Cell Cycle.

[34] Coley H.M., Safuwan N.A.M., Chivers P., Papacharalbous E., Giannopoulos T., Butler-Manuel S., Madhuri K., Lovell D.P., Crook T. (2012). The cyclin-dependent kinase inhibitor p57Kip2 is epigenetically regulated in carboplatin resistance and results in collateral sensitivity to the CDK inhibitor seliciclib in ovarian cancer. Br. J. Cancer.

[35] Alam S., Sultana A., Wang G., Mollah N.H. (2022). Gene expression profile analysis to discover molecular signatures for early diagnosis and therapies of triple-negative breast cancer. Front. Mol. Biosci..

[36] Packeiser E.-M., Engels L., Nolte I., Goericke-Pesch S., Escobar H.M. (2023). MDR1 inhibition reverses doxorubicin-resistance in six doxorubicin-resistant canine prostate and bladder cancer cell lines. Int. J. Mol. Sci..

[37] Nicoś M., Rolska-Kopińska A., Krawczyk P., Grenda A., Bożyk A., Szczyrek M., Milanowski J. (2021). Effect of TOP2A and ERCC1 gene polymorphisms on the efficacy and toxicity of cisplatin and etoposide-based chemotherapy in small cell lung cancer patients. Arch. Med. Sci..

[38] Lee K.C., Bramley R.L., Cowell I.G., Jackson G.H., Austin C.A. (2016). Proteasomal inhibition potentiates drugs targeting DNA topoisomerase II. Biochem. Pharmacol..

[39] Saleh A.M., Solayman M., Hoque M.M., Khan M.A.K., Sarwar M.G., Halim M.A. (2016). Inhibition of DNA topoisomerase type IIα (TOP2A) by mitoxantrone and its halogenated derivatives: A combined density functional and molecular docking study. BioMed Res. Int..

[40] Wang Z., Zhu Q., Li X., Ren X., Li J., Zhang Y., Zeng S., Xu L., Dong X., Zhai B. (2022). TOP2A inhibition reverses drug resistance of hepatocellular carcinoma to regorafenib. Am. J. Cancer Res..

[41] Caputo W.L., de Souza M.C., Basso C.R., Pedrosa V.d.A., Seiva F.R.F. (2023). Comprehensive Profiling and Therapeutic Insights into Differentially Expressed Genes in Hepatocellular Carcinoma. Cancers.

[42] Parker L.P., Taylor D.D., Kesterson S., Gercel-Taylor C. (2009). Gene expression profiling in response to estradiol and genistein in ovarian cancer cells. Cancer Genom. Proteom..

[43] Kloskowski T., Szeliski K., Fekner Z., Rasmus M., Dąbrowski P., Wolska A., Siedlecka N., Adamowicz J., Drewa T., Pokrywczyńska M. (2021). Ciprofloxacin and levofloxacin as potential drugs in genitourinary cancer treatment—The effect of dose–Response on 2D and 3D cell cultures. Int. J. Mol. Sci..

[44] Singh J., Srivastva A.K., Mandal P., Chandra S., Dubey D., Dwivedi A., Chopra D., Tripathi A., Ray R.S. (2018). Under ambient UVA exposure, pefloxacin exhibits both immunomodulatory and genotoxic effects via multiple mechanisms. J. Photochem. Photobiol. B Biol..

[45] Yan C., Niu Y., Wang X. (2022). Blood transcriptome analysis revealed the crosstalk between COVID-19 and HIV. Front. Immunol..

[46] Nair S.V.G., Ziaullah, Rupasinghe H.P.V. (2014). Fatty acid esters of phloridzin induce apoptosis of human liver cancer cells through altered gene expression. PLoS ONE.

[47] Zhu J., Lin S., Zou X., Chen X., Liu Y., Yang X., Gao J., Zhu H. (2023). Mechanisms of autophagy and endoplasmic reticulum stress in the reversal of platinum resistance of epithelial ovarian cancer cells by naringin. Mol. Biol. Rep..

[48] Jia L., Gao H. (2022). Machine Learning for in silico ADMET prediction. Artificial Intelligence in Drug Design.

[49] Yang H., Lou C., Sun L., Li J., Cai Y., Wang Z., Li W., Liu G., Tang Y. (2019). admetSAR 2.0: Web-service for prediction and optimization of chemical ADMET properties. Bioinformatics.

[50] Barrett T., Wilhite S.E., Ledoux P., Evangelista C., Kim I.F., Tomashevsky M., Marshall K.A., Phillippy K.H., Sherman P.M., Holko M. (2013). NCBI GEO: Archive for functional genomics data sets—Update. Nucleic Acids Res..

[51] Wilson R. (2023). The application of capability indices in the validation of ELISA methodology. Bioanalysis.

[52] Szklarczyk D., Kirsch R., Koutrouli M., Nastou K., Mehryary F., Hachilif R., Gable A.L., Fang T., Doncheva N.T., Pyysalo S. (2023). The STRING database in 2023: Protein–protein association networks and functional enrichment analyses for any sequenced genome of interest. Nucleic Acids Res..

[53] Shannon P., Markiel A., Ozier O., Baliga N.S., Wang J.T., Ramage D., Amin N., Schwikowski B., Ideker T. (2003). Cytoscape: A software environment for integrated models of Biomolecular Interaction Networks. Genome Res..

[54] Bartha Á., Győrffy B. (2021). TNMplot.com: A web tool for the comparison of gene expression in normal, tumor and metastatic tissues. Int. J. Mol. Sci..

[55] Burley S.K., Bhikadiya C., Bi C., Bittrich S., Chao H., Chen L., Craig P.A., Crichlow G.V., Dalenberg K., Duarte J.M. (2023). RCSB Protein Data Bank (RCSB.org): Delivery of experimentally-determined PDB structures alongside one million computed structure models of proteins from artificial intelligence/machine learning. Nucleic Acids Res..

[56] Hatami S., Sirous H., Mahnam K., Najafipour A., Fassihi A. (2023). Preparing a database of corrected protein structures important in cell signaling pathways. Res. Pharm. Sci..

[57] Farahmand S., SamadiAfshar S., HajiHosseini R., Babari T. (2025). Carnosic Acid as a Potent Ag85C Inhibitor Identified Through Integrated Pharmacokinetic Evaluation and Molecular Modeling in Mycobacterium Tuberculosis Drug Discovery. J. Pharm. Innov..

[58] Yang J., Roy A., Zhang Y. (2013). Protein–ligand binding site recognition using complementary binding-specific substructure comparison and sequence profile alignment. Bioinformatics.

[59] Handsel J., Matthews B., Knight N.J., Coles S.J. (2021). Translating the InChI: Adapting neural machine translation to predict IUPAC names from a chemical identifier. J. Cheminform..

[60] Eberhardt J., Santos-Martins D., Tillack A.F., Forli S. (2021). AutoDock Vina 1.2.0: New docking methods, expanded force field, and python bindings. J. Chem. Inf. Model..

[61] Samadiafshar S., Farahmand S., Nik-Akhtar A., Garmsiri N., Garmsiri F., Samadiafshar S., Azizi R. (2025). Antibacterial and Phytochemical Properties of Stachys schtschegleevii Oil Extract: Investigating Interaction and Antimicrobial Activity against Urinary Tract Infection Bacteria Through In Silico and In Vitro. Iran. J. Anal. Chem..

[62] Dallakyan S., Olson A.J. (2014). Small-molecule library screening by docking with PyRx. Chemical Biology: Methods and Protocols.

[63] Adasme M.F., Linnemann K.L., Bolz S.N., Kaiser F., Salentin S., Haupt V.J., Schroeder M. (2021). PLIP 2021: Expanding the scope of the protein–ligand interaction profiler to DNA and RNA. Nucleic Acids Res..

[64] SamadiAfshar S., Azizi H., Masoudi M., SamadiAfshar S., Nikakhtar A., Skutella T. (2026). Preoperative differentiation of borderline and malignant ovarian tumors using interpretable machine learning. J. Ovarian Res..

[65] Farahmand S., SamadiAfshar S., Khalili M., Hosseini R.H. (2025). Therapeutic implications of Epirubicin-induced miRNA-22 and miRNA-331 upregulation on cell viability and metastatic potential in triple-negative breast cancer. Hum. Gene.

[66] Farahmand S., SamadiAfshar S., Hosseini L. (2024). TA-Cloning for Diabetes Treatment: Expressing Corynebacterium Malic Enzyme Gene in *E. coli*. Curr. Microbiol..

