# Supplementary material for: Multifaceted Integrated Analysis of CDK1 and TOP2A Signaling Pathways for Multi-Target Therapeutic Intervention in Epithelial Ovarian Cancer
Source: Int J Mol Sci. 2026 Jun 10;27(12):5264. doi: 10.3390/ijms27125264 (PMC13300235; doi:10.3390/ijms27125264)
Supplement: Supplementary file 1 [file ijms-27-05264-s001.zip › ijms-4307879-supplementary.pdf]

Multifaceted Integrated Analysis of CDK1 and TOP2A Signaling Pathways for Multi-Target Therapeutic Intervention in Epithelial Ovarian Cancer

Table S1. The results of 2D and 3D molecular docking interactions between the proteins CDK1 and TOP2A and 29 potential drugs

Protein / Ligand	CDK1		TOP2A	
	3D	2D	3D	2D
**Alsterpaullone**	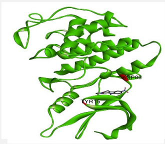	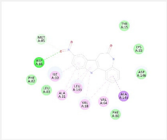	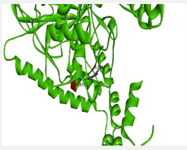	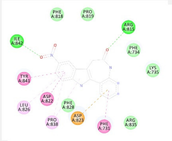
**Avotaciclib**	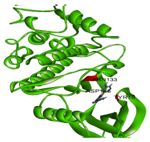	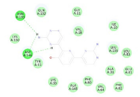	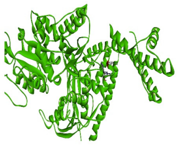	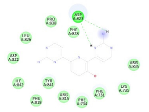
**Fostamatinib**	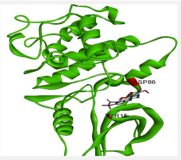	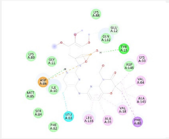	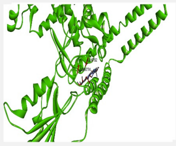	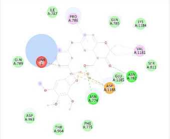
**Naringin**	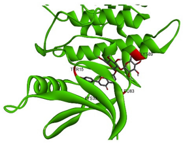	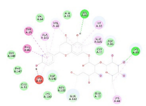	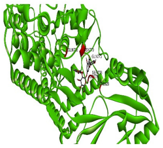	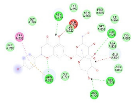
**Amsacrine**	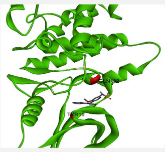	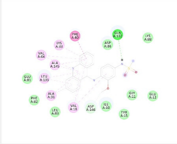	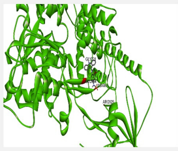	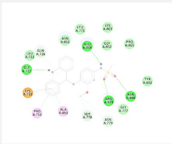
**Dexrazoxane**	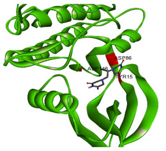	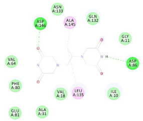	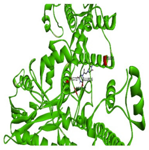	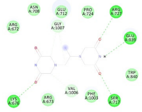
**Valrubicin**	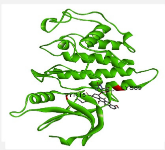	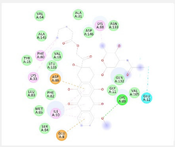	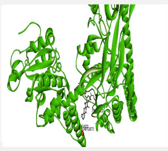	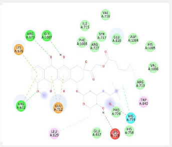
**Teniposide**	*	*	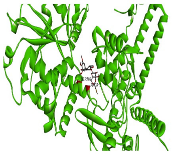	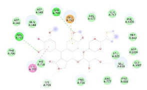

Continue Table S1

Protein / Ligand	CDK1		TOP2A	
	3D	2D	3D	2D
**Etoposide**	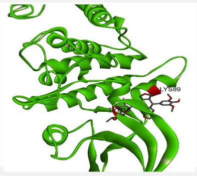	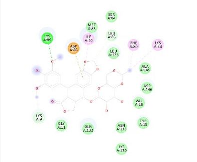	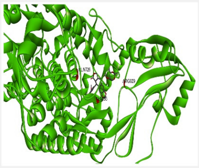	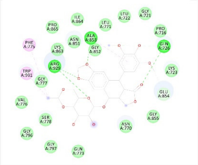
**Doxorubicin**	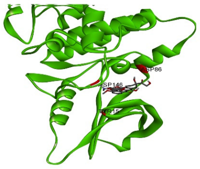	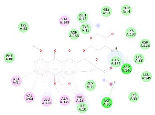	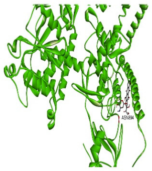	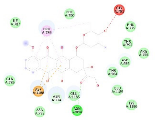
**Idarubicin**	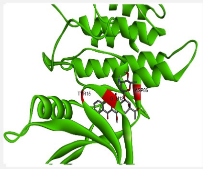	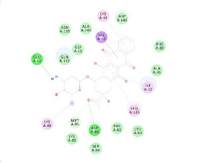	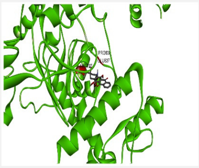	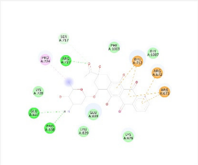
**Mitoxantrone**	*	*	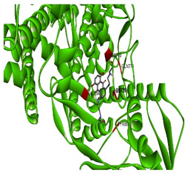	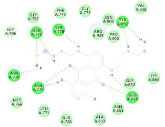
**Epirubicin**	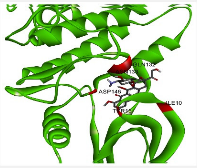	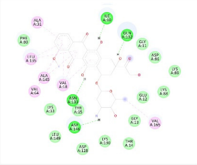	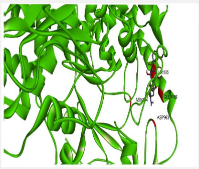	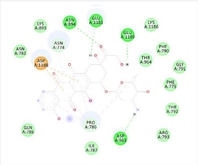
**Podofilox**	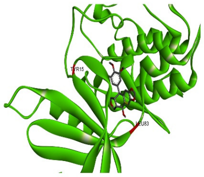	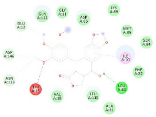	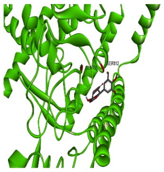	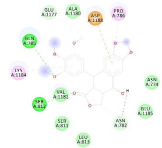
**Genistein**	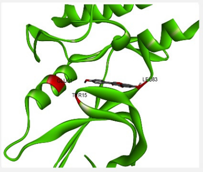	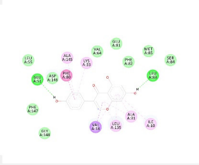	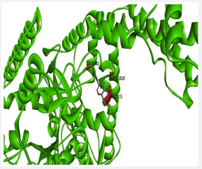	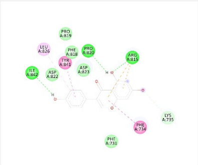
**Ciprofloxacin**	*	*	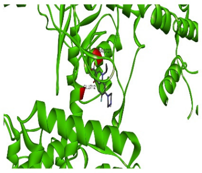	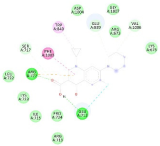

Continue Table S1

Protein / Ligand	CDK1		TOP2A	
	3D	2D	3D	2D
**Enoxacin**	*	*	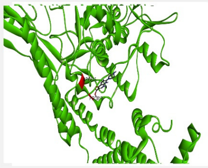	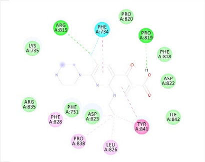
**Fleroxacin**	*	*	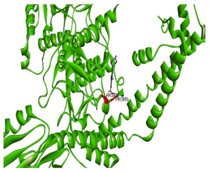	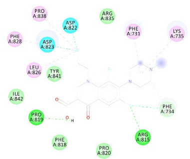
**Lomefloxacin**	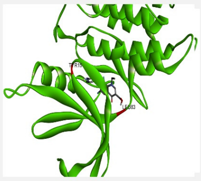	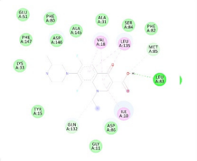	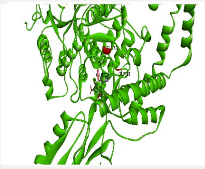	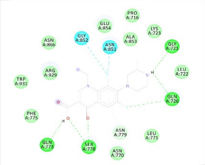
**Moxifloxacin**	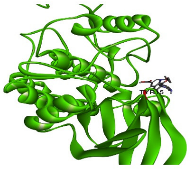	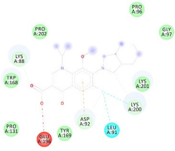	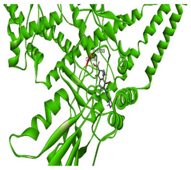	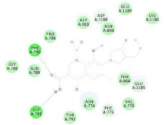
**Norfloxacin**	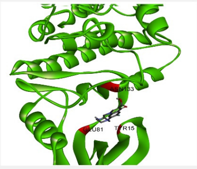	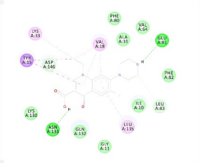	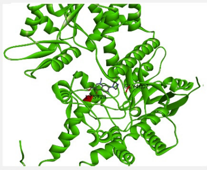	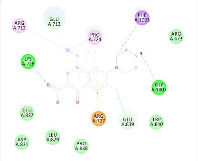
**Pefloxacin**	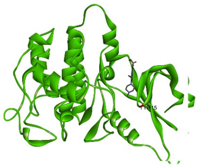	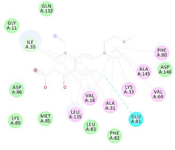	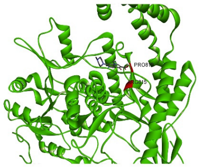	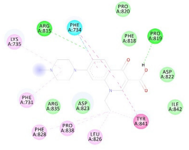
**Sparfloxacin**	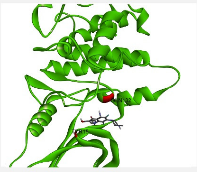	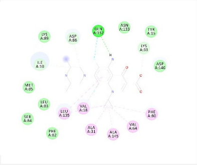	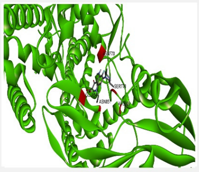	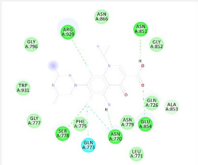
**Trovafloxacin**	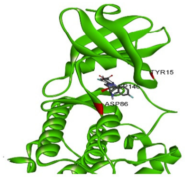	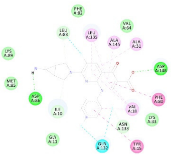	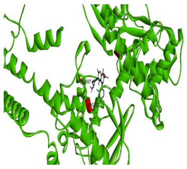	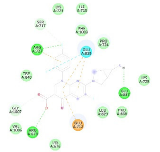

Continue Table S1

Protein / Ligand	CDK1		TOP2A	
	3D	2D	3D	2D
**Ofloxacin**	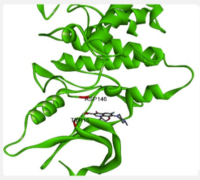	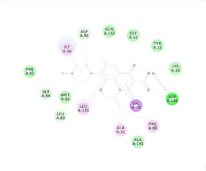	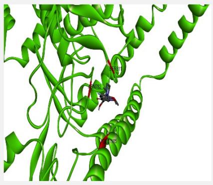	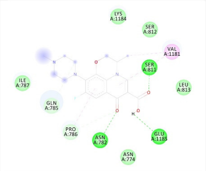
**Lucanthone**	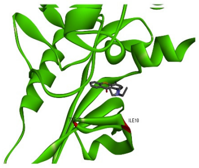	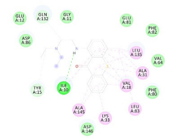	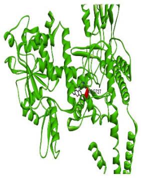	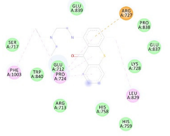
**Daunorubicin**	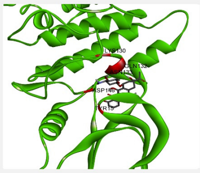	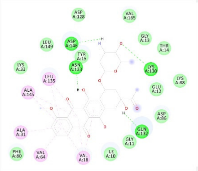	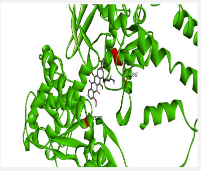	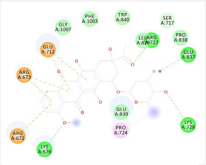
**Finafloxacin**	*	*	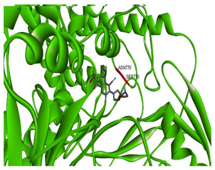	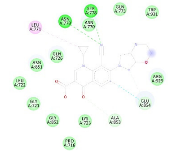
**Aldoxorubicin**	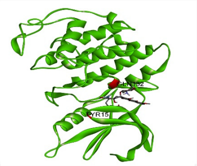	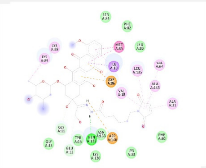	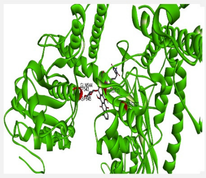	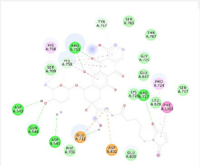
